# Improving the Outcome of Brain-Injured Patients by Non-Continuous Feeding to Prevent Dysbiosis

**DOI:** 10.3390/nu18172907

**Published:** 2026-09-04

**Authors:** Alberto Corriero, Rossana Soloperto, Mariateresa Giglio, Fabio Silvio Taccone, Filomena Puntillo, Jean-Charles Preiser

**Affiliations:** 1 Department of Interdisciplinary Medicine—ICU Section, University of Bari Aldo Moro, 70124 Bari, Italy; rossana.soloperto@gmail.com (R.S.); mariateresa.giglio@uniba.it (M.G.); filomena.puntillo@uniba.it (F.P.); 2 Department of Intensive Care, Université Libre de Bruxelles (ULB), 1070 Brussels, Belgium; fabio.taccone@ulb.be; 3 Department of Internal Medicine, Institut Jules Bordet, Université Libre de Bruxelles (ULB), 1070 Brussels, Belgium

**Keywords:** intermittent fasting, gastrointestinal microbiome, brain–gut axis, dysbiosis, neuroinflammatory diseases, sepsis-associated encephalopathy, brain injuries, traumatic, enteral nutrition, critical illness

## Abstract

*Background*: Acute brain injury, including traumatic brain injury (TBI), stroke, subarachnoid hemorrhage and secondary neurological injury, such as sepsis-associated encephalopathy (SAE) and delirium, is a major cause of morbidity in the intensive care unit (ICU), and few treatments alter its course once it is established. Critical illness, and brain injury in particular, rapidly disrupts the gut microbiome (GM) and the production of its metabolites. This dysbiosis matters most in neurologically injured patients, because microbial metabolites and a leaky intestinal barrier feed neuroinflammation through the gut–brain axis. Feeding timing and fasting affect circadian biology, the daily rhythm of the GM, and host metabolic pathways, such as ketogenesis, insulin signaling and autophagy. This review asks whether time-restricted or fasting-mimicking strategies can preserve these functions and reduce neuroimmune dysregulation after brain injury. *Methods*: In this narrative review, we searched the literature for randomized trials, crossover pilot studies, mechanistic human research, and guideline statements comparing continuous, cyclic, and intermittent enteral strategies, and evaluated translational pathways for GM-targeted feeding interventions. *Results*: Available studies showed no consistent difference in mortality between continuous and intermittent gastric feeding in ICU adults on mechanical ventilation, although the largest pooled analyses report more diarrhea, more abdominal distension and longer ICU stay with intermittent schedules, most pronounced in ventilated patients. While intermittent/cyclic regimens were not associated with an improvement of patient-centered outcomes, pilot studies demonstrated that short macronutrient interruptions (e.g., 12 h) reliably induced a metabolic fasting response in patients with a prolonged ICU stay. One randomized controlled trial (RCT) had GM end-points and reported feasible modulation of gut taxa and 58 differentially abundant serum metabolites with sequential (continuous-to-intermittent) feeding. Only five studies enrolled dedicated brain-injured cohorts; none was designed around neurological or gut–brain mechanistic outcomes, and none measured GM or neuroinflammation. *Conclusions*: Time-restricted, microbiome-protecting approaches are biologically plausible and deserve a phased translational program to test them in clinical trials. Future trials should focus on brain-injured and other neurologically relevant ICU patients, in whom the gut–brain axis is most engaged, and should pair mechanistic endpoints with clinical safety and proper control for confounders. No clinical study has yet tested whether non-continuous feeding alters neuroinflammatory or neurological outcomes after acute brain injury. There is currently no direct evidence supporting the effectiveness of this intervention in this population, and the case made here is mechanistic, not empirical.

## 1. Introduction

Acute brain injury is common and severe in critical care. It may be primary, as in traumatic brain injury (TBI), aneurysmal subarachnoid hemorrhage, intracerebral hemorrhage and ischemic stroke, or secondary, as in sepsis-associated encephalopathy (SAE) and intensive care unit (ICU)-associated delirium [1]. These patients often need prolonged mechanical ventilation and long ICU and hospital stays, and many are left with cognitive, functional or psychiatric impairment that lasts well beyond discharge [2]. This post-intensive care syndrome therefore identifies new or worsening impairments in physical, cognitive or mental health that persist after critical illness and beyond hospital discharge [3]. These long-term sequelae lower survivors’ quality of life and raise health-care costs through prolonged rehabilitation and repeated hospital readmission [4,5]. Survivors from brain injury are among the most affected; their course is frequently complicated by secondary insults, such as systemic inflammation, nosocomial infection, dysglycemia and organ dysfunction, which aggravate the primary lesion and worsen the outcome [6]. The presence of neuroinflammation has been linked to adverse neurological outcomes, including the development of delirium [7] and SAE, as well as secondary injury following major trauma [8,9]. This connection is thought to be partly mediated by the microbiota–gut–brain axis, a network of immune, neural, endocrine, and metabolic pathways through which intestinal disturbances directly influence central nervous system (CNS) function. Feeding and nutrition significantly influence these pathways, as nutrients, microbial metabolites, and gut-derived immune signals interact with systemic and neural circuits that regulate inflammation and neuro-immune responses [10]. Critical illness itself is a primary driver of CNS dysfunction, as it induces gut dysbiosis, a condition in which beneficial commensals are lost, and opportunistic pathogens proliferate. This affects the intestinal barrier function, the production of local metabolites, and triggers systemic inflammation, immune dysregulation, and metabolic dysfunction, affecting distant organs, including the brain [11]. The gut microbiome (GM) and its associated genes configure the gut as both a biomarker and a mediator of pathophysiology in the ICU, leading to immune dysregulation, metabolic disruption, and secondary organ dysfunction [12].

Once the primary brain insult has occurred, therapeutic options capable of substantially modifying the trajectory of secondary brain injury remain limited. Consequently, increasing attention has shifted toward systemic, potentially modifiable factors that influence neuroinflammation, immune homeostasis, and neurological recovery. Among these, nutritional strategies and the GM have emerged as promising therapeutic targets, although their clinical efficacy has yet to be established in adequately designed interventional studies [13]. In this review, we examine the mechanisms through which critical illness and acute brain injury disrupt the GM and discuss the bidirectional interactions within the gut–brain axis. We then critically appraise the available evidence comparing continuous, intermittent, cyclic, and fasting-mimicking enteral feeding strategies, and propose a conceptual framework for evaluating microbiome-preserving nutritional interventions in patients with acute neurological injury. No published trial has measured microbiome or neuroinflammatory endpoints under different feeding schedules in patients with acute brain injury, and no direct clinical evidence of effectiveness is therefore available in this population. What follows is an appraisal of biological rationale and of trial feasibility, not of demonstrated efficacy. This is a narrative review. The literature search reported in Section 7 was structured and is described in full for transparency, but it was not prospectively registered and does not claim the exhaustive coverage of a systematic review.

## 2. Dysbiosis

As soon as the patient is admitted into the ICU, the GM undergoes a profound and rapid disruption of its “steady state”, i.e., eubiosis, shifting from a microbiome to a “pathobiota”, the so called dysbiosis, which can happen in as little as 6 h and is increasingly recognized as a key factor in the progression of organ failure and poor outcomes [11].

Specific alterations in the GM have been described as a reduced microbial richness and diversity, marked by increased opportunistic and nosocomial pathogens, such as *Enterococcus* and *Klebsiella*, and a depletion of beneficial commensal bacteria, including *Lachnospiraceae* and *Ruminococcaceae* [14]. This microbial rearrangement is driven by various agents that typically occur in the ICU, including broad-spectrum antibiotics, especially those that do not spare anaerobic populations, proton pump inhibitors, and different approaches to enteral nutrition (EN) itself [11]. Acute brain injury introduces additional mechanisms of gut dysfunction that extend beyond those associated with critical illness alone. The early catecholamine surge and disruption of central autonomic regulation impair splanchnic perfusion, gastrointestinal motility, epithelial barrier integrity, and mucosal immune homeostasis, creating a permissive environment for alterations in microbial ecology [13,15]. Neurocritical care interventions, including sedation, vasoactive drugs, mechanical ventilation, and frequently reduced enteral nutrient delivery, may further amplify these disturbances by limiting mesenteric perfusion and slowing intestinal transit [9,16]. Collectively, these neurogenic and treatment-related factors may promote a brain-injury-specific pattern of dysbiosis; however, whether such a microbial phenotype is distinct from that of the broader ICU population has yet to be systematically defined.

The impairment of this microbial “symphony” [17] probably has significant consequences that extend beyond the gut. Dysbiosis also impairs several metabolic pathways, including primary bile acid synthesis, which in turn exacerbates intestinal barrier dysfunction [18]. Ultimately, the breakdown of the gut barrier worsens the critical illness and sets the premises for a multi-organ dysfunction syndrome (MODS) to take place and develop abruptly [19]. Clinically, this breakdown translates into common ICU complications. For instance, enteral nutrition-related diarrhea (END) is strongly associated with severe dysbiosis, marked by significantly lower bacterial diversity and the overgrowth of specific pathogens [14]. On the contrary, the ability to tolerate enteral feeding appears to be linked to distinct microbiome profiles and to the levels of short-chain fatty acids (SCFAs) early in the course of nutrition therapy [20], which paves the way for research into microbial markers that could predict intolerance [21].

## 3. Neuroinflammation

In critically ill and brain-injured patients, neuroinflammation usually appears as acute brain dysfunction, from mild delirium to SAE, and it adds to the cognitive impairment that many survivors carry for months after discharge [22]. The more severe and prolonged this dysfunction is, the longer patients stay in the ICU and the higher their mortality [23]. There is still no specific diagnostic test, but blood and cerebrospinal markers of glial and neuronal injury such as glial fibrillary acidic protein (GFAP), neurofilament light chain (NfL) and S100B, together with pro-inflammatory cytokines, rise as the encephalopathy worsens and predict a poorer neurological outcome [24,25]. These clinical changes reflect the activation of the resident immune cells of the central nervous system, mainly microglia.

Microglial cells in their homeostatic state act as sentinels, eliminating superfluous synapses and supporting neuronal survival. When activated, they polarize into a pro-inflammatory M1 phenotype, releasing cytokines, reactive oxygen species (ROS), and nitric oxide (NO) that drive neuroinflammation, or into an anti-inflammatory M2 phenotype, promoting repair and neuroprotection through interleukin-10 (IL-10), transforming growth factor-beta (TGF-β), and trophic factors. Beyond this M1/M2 dichotomy, disease-, age-, and context-specific phenotypes have emerged, reflecting neuroplasticity [26].

Therefore, neuroinflammation is not a bystander phenomenon but part of the injury itself. Necrotic tissue releases damage-associated molecular patterns that engage pattern-recognition receptors on microglia and astrocytes. Mechanical and ischemic damage opens the blood–brain barrier and allows circulating monocytes and neutrophils to enter the parenchyma and amplify the resident glial response [6]. The consequences are not confined to the injured brain, since the same inflammatory signaling reaches the gut and disturbs motility, barrier function and microbial composition [13]. Edema, impaired cerebral autoregulation and recurrent secondary insults sustain this loop, so that glial activation persists for days to weeks rather than hours. The extent of the final lesion consequentially depends not only on the initial impact but on the intensity and duration of the response that follows it.

This response leaves measurable traces at the bedside, although the available markers report glial and neuronal injury rather than the inflammatory process itself. In the TRACK-TBI cohort, plasma GFAP and UCH-L1 measured on the day of injury predicted death at six months with areas under the curve of 0.87 and 0.89. They also added prognostic information to established clinical models in patients presenting with a Glasgow Coma Scale of 3–12 [27]. In CENTER-TBI, the same class of glial and neuronal markers improved outcome prediction across the range of imaging severities [28]. In a further CENTER-TBI analysis, the first week of intensive care was modeled as a set of disease trajectories in 1728 patients with traumatic brain injury. The variables that separated those trajectories most strongly were these same glial and neuronal biomarkers, together with the daily swing between the highest and the lowest blood glucose [29], an observation that places metabolic instability, and not the injury alone, among the factors that track how these patients evolve.

## 4. Potential Links Between Dysbiosis and Neuroinflammation

The gut–brain axis is a bidirectional communication system that comprises the gut, the CNS, the sympathetic and parasympathetic nervous systems, and the hypothalamic–pituitary–adrenal (HPA) axis [30].

The link between GM composition and neurological outcomes in critical illness have been reported predominantly in cohorts with primary brain injury and in patients with sepsis, rather than in undifferentiated ICU populations. Observational clinical studies of acute neurological injury (TBI, intracerebral hemorrhage and stroke) and of neurologic ICU populations show disease-specific gut signatures (for example, enrichment of *Enterobacteriaceae* in some TBI cohorts and altered GM composition after intracerebral hemorrhage) and link dysbiosis to worse neuro-inflammation or delayed recovery in exploratory studies [31,32,33]. However, antibiotic exposure, which is highly prevalent in these cohorts and a dominant driver of dysbiosis, is variably reported (class, timing, and cumulative days are often missing or inconsistent), limiting causal inference in human datasets [34]. Translational and animal models point to a causal role for the GM in post-injury neuroinflammation and recovery. Antibiotic-induced dysbiosis before or after experimental TBI altered lesion size, microglial activation, and behavioral outcomes, indicating that perturbations of the GM can both worsen and (in some protocols) improve neuroinflammatory injury, depending on timing and method of manipulation [35,36].

### 4.1. Microbiome–Gut–Brain Signaling Routes

Dysbiosis can modulate neuroinflammation through several mechanisms.

#### 4.1.1. Short-Chain Fatty Acids

During critical illness, the production of SCFAs by GM is decreased as reflected by low fecal concentrations. SCFAs (butyrate, acetate, propionate) produced by saccharolytic commensals from dietary fibers influence neuroimmune processes, ranging from the regulation of microglia to the maintenance of the blood–brain barrier (BBB) integrity and neuroimmune homeostasis [37]. SCFAs are involved in the regulation of intestinal motility, immunity, and energy balance, and have receptors in various systems throughout the body, including the central and peripheral nervous systems [20,38,39]. Hence, SCFAs can be considered as central mediators linking feeding patterns to brain immune status via energy metabolism, mucus production, tight-junction integrity, and reprogramming the immune system [40], which in turn affects the dynamic spectrum of microglial phenotypes and neurotrophic signaling. More specifically, SCFAs such as butyrate act on host cells through three routes: binding to free fatty acid receptors 2 and 3 (FFAR2/FFAR3), inhibiting histone deacetylases with related epigenetic effects, and entering cells through monocarboxylate transporters. Together these actions suppress nuclear factor kappa B (NF-κB) and inflammasome signaling in endothelial and myeloid cells, stabilize BBB function [41] and modulate the production of central cytokines [42].

SCFA supplementation in an experimental model has been shown to improve cognitive outcomes, including spatial learning, following injury [39]. This suggests a potential path from gut dysbiosis to brain dysfunction in sepsis and other ICU syndromes.

#### 4.1.2. Inflammasome Activation

Dysbiosis-derived danger signals activate the NOD-, LRR- and pyrin domain-containing protein 3 (NLRP3) inflammasome and promote IL-1β and IL-18 release, contributing to neuroinflammation and pyroptosis. Assembly requires two signals: a priming step in which pattern-recognition receptors drive NF-κB-dependent transcription of NLRP3 and pro-IL-1β, and an activation step in which potassium efflux and mitochondrial stress trigger oligomerization with ASC and caspase-1, which then matures both cytokines and cleaves gasdermin D to form the release pore. This axis has been described in white matter injury after intracerebral hemorrhage, one of the few acute brain-injury settings in which it has been examined directly [43]. GM-derived indole-3-propionic acid can inhibit this pathway, linking microbial composition to neuroimmune modulation [44,45]. More specifically this pathway has been associated with multiple sclerosis, Alzheimer’s disease, Parkinson’s disease, neuropsychiatric diseases, and sepsis [11].

#### 4.1.3. Bile Acids and Bile-Acid Receptor Signaling

Gut bacteria convert primary bile acids into secondary bile acids that act on the farnesoid X receptor (FXR) and Takeda G-protein receptor 5 (TGR5) and help regulate systemic and central immune responses; when dysbiosis depletes these bacteria the signal is lost, which can worsen neuroinflammation [43]. In the ileum, bile-acid activation of FXR induces the enterokine fibroblast growth factor 15 in rodents and its human orthologue FGF19, which feeds back on hepatic bile-acid synthesis and contributes to bile-acid and glucose homeostasis across the transition from the fed to the fasted state [46]. Secondary bile acids also reach the central nervous system across the blood–brain barrier, where TGR5 is expressed [47]. In a murine model of ischemic stroke, microbiota-derived ursodeoxycholic acid was depleted, and restoring it suppressed NLRP3-dependent cytokine release from microglia through TGR5-PKA signaling and reduced infarct size [48].

#### 4.1.4. Tryptophan–Indole–Kynurenine Metabolism

Bacterial indoles and kynurenine-pathway metabolites bind the aryl hydrocarbon receptor and influence how reactive microglia become and whether the mediators they produce protect or harm neurons [43]. Three routes compete for dietary tryptophan: host indoleamine 2,3-dioxygenase and hepatic tryptophan 2,3-dioxygenase supply kynurenines, bacterial tryptophanase supplies indoles, and tryptophan hydroxylase 1 in enterochromaffin cells supplies serotonin. Indole, indoxyl-3-sulfate, indole-3-propionic acid and indole-3-aldehyde act on AhR in astrocytes and, together with type I interferon signaling, restrain the transcriptional programs that sustain central nervous system inflammation. Antibiotic depletion of the microbiota worsens experimental autoimmune encephalomyelitis and supplying these metabolites reverses it [49]. The same metabolites act on microglia, which govern astrocyte behavior through TGF-α and VEGF-B [50]. Circulating AhR agonists are reduced in multiple sclerosis, the condition in which this axis has been characterized most fully; whether it behaves comparably in acute brain injury has not been tested.

#### 4.1.5. Vagal (Cholinergic) Anti-Inflammatory Reflex

Microbial signals act on vagal afferents and the cholinergic anti-inflammatory pathway, a direct neural route through which the gut can dampen or amplify brain inflammation [43]. Afferent fibers in the gut wall sense microbial metabolites and enteroendocrine mediators and project to the nucleus of the solitary tract, while efferent vagal activity engages the splenic sympathetic nerve and acetylcholine-producing lymphocytes, whose acetylcholine acts on α7 nicotinic receptors on macrophages to suppress NF-κB-driven cytokine release [51]. Loss of vagal tone therefore removes a brake on cytokine production rather than merely a signal.

#### 4.1.6. Gut Barrier Integrity and Endotoxin Translocation

Increased intestinal permeability allows the translocation of endotoxins such as lipopolysaccharide (LPS), which then enter the systemic circulation and reach the cerebral vasculature, where they activate microglia and the cerebral endothelium mainly through Toll-like receptor 4 (TLR4). In this way gut-derived LPS can contribute to blood–brain barrier breakdown and central inflammation [52]. The same signaling downregulates occludin and the claudins, the tight-junction proteins whose loss opens the paracellular route. LPS is delivered to its receptor by LPS-binding protein and CD14, and the MD-2–TLR4 complex then activates NF-κB, inducing the adhesion molecules and cytokines that degrade the endothelial junctions of the barrier itself [53].

#### 4.1.7. HPA Axis and Systemic Stress Response

GM-derived signals also modulate the HPA axis and systemic stress responses, contributing to neuroimmune homeostasis [26,54]. Gut-derived metabolites and cytokines influence hypothalamic corticotropin-releasing hormone release and the downstream ACTH and cortisol response, while glucocorticoids in turn shape microglial reactivity and intestinal permeability, closing a bidirectional loop [30].

**Figure 1:**
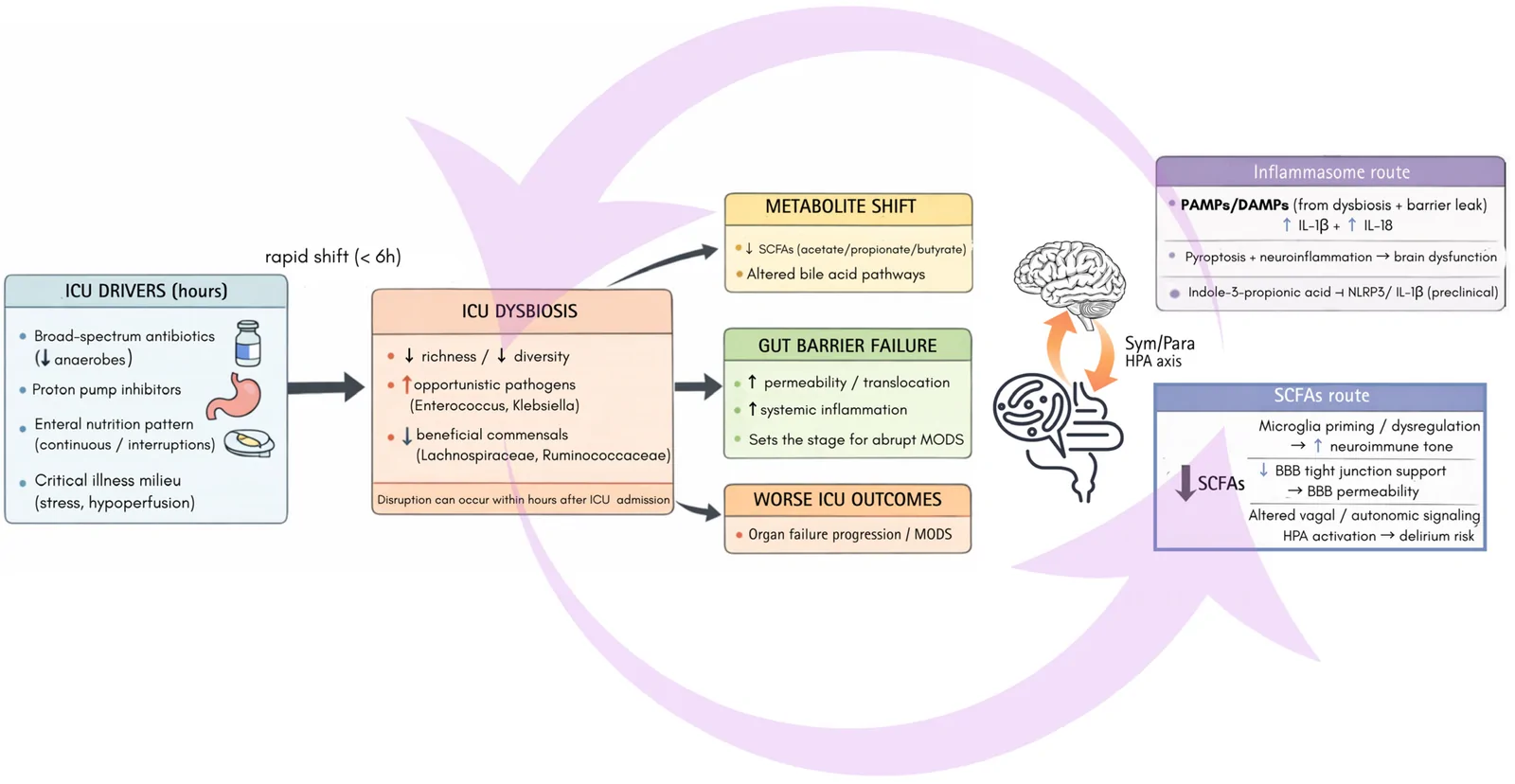
Schematic representation of ICU-associated gut dysbiosis and downstream effects on metabolism, barrier integrity, systemic inflammation, and the gut–brain axis. ICU, intensive care unit; SCFAs, short-chain fatty acids; BBB, blood–brain barrier; HPA axis, hypothalamic–pituitary–adrenal axis; PAMPs, pathogen-associated molecular patterns; DAMPs, damage-associated molecular patterns; NLRP3, NOD-, LRR- and pyrin domain-containing protein 3; IL, interleukin; MODS, multi-organ dysfunction syndrome. The pathways depicted are mechanistic and derive largely from preclinical data; they are not established in acute brain injury.

The GM is increasingly viewed as an active regulator of neuroinflammation rather than a passive bystander. Through microbial metabolites, immune signaling, and neural communication along the gut–brain axis, it modulates microglial development, activation, and functional polarization, thereby influencing the equilibrium between tissue injury and repair. Experimental studies further demonstrate that microbial perturbations affect cerebral transcriptional programs, synaptic plasticity, and neuroimmune signaling in brain regions critical for cognition and executive function, including the hippocampus and prefrontal cortex. However, these mechanistic insights originate predominantly from germ-free animals, microbiome manipulation experiments, or models of chronic neurological disease. Direct evidence that similar pathways operate in patients with acute brain injury remains limited. The downstream consequences of ICU-associated dysbiosis on gut barrier integrity, systemic inflammation, and the gut–brain axis are summarized in Figure 1.

### 4.2. Feeding–Fasting Cycles: Mechanistic Links

**Figure 2:**
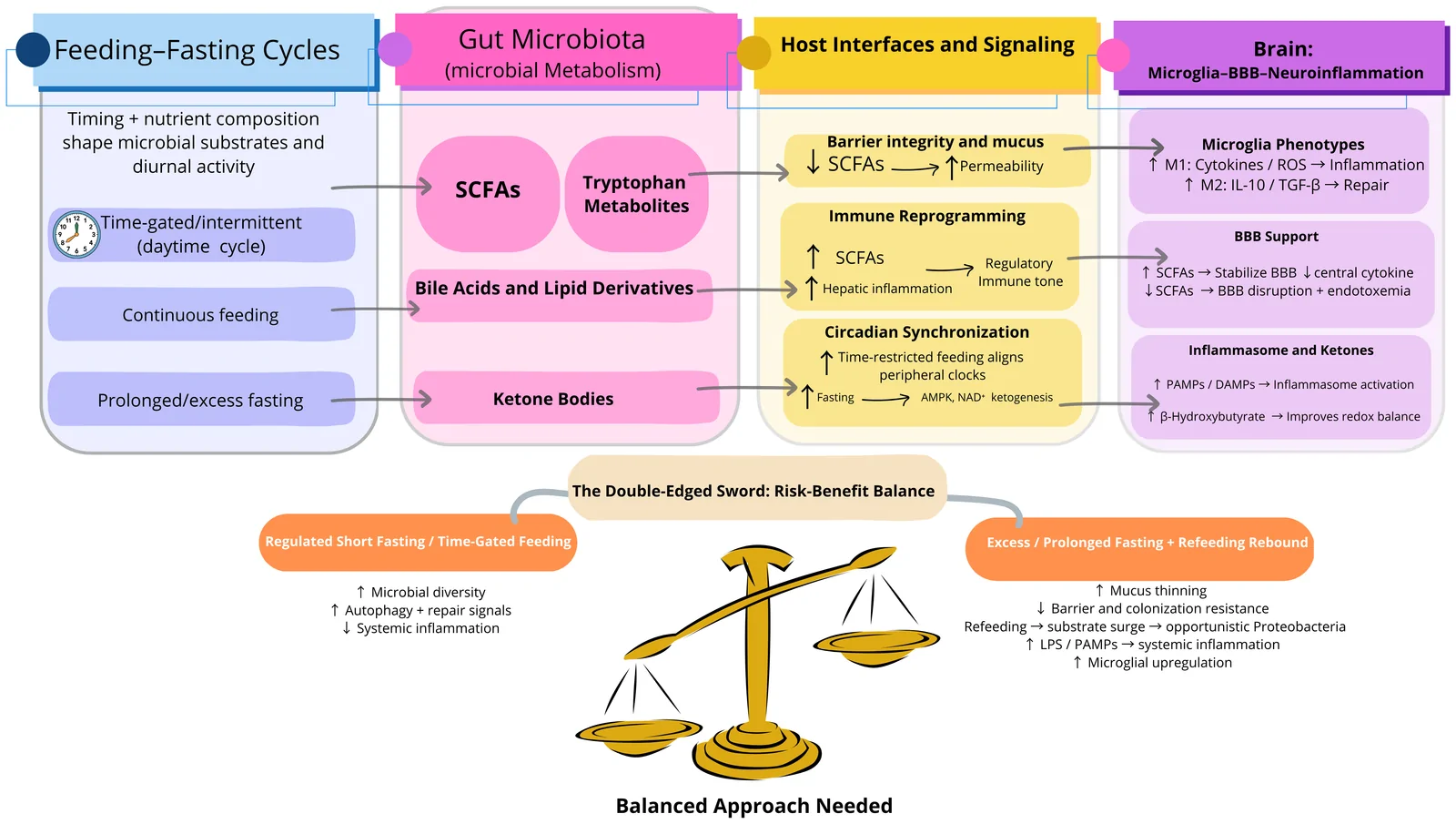
Mechanistic links between feeding–fasting cycles, gut microbial metabolism, host signaling, and brain inflammation. Time-restricted feeding supports SCFA production, barrier integrity, immune tone, and circadian alignment. Excess fasting and refeeding can disrupt mucus, favor LPS/PAMP translocation, and promote neuroinflammation. Abbreviations: SCFAs, short-chain fatty acids; LPS, lipopolysaccharide; PAMPs, pathogen-associated molecular patterns; BBB, blood–brain barrier. The pathways depicted are mechanistic and derive largely from preclinical data; the circadian arm concerns peripheral clocks rather than central suprachiasmatic rhythmicity.

Feeding timing and nutrient composition act on the network summarized in Figure 2.

Ultimately, it appears that feeding fasting cycles are one of the key factors that can significantly influence SCFA production, as the timing and composition of the feeding are crucial [18]. Cyclic or intermittent feeding maintains the physiological oscillations in substrate availability that synchronize microbial metabolism with host circadian biology, thereby preserving SCFA production and other microbiome-derived signaling pathways. By contrast, continuous nutrient infusion attenuates these temporal fluctuations, while prolonged fasting or absence of fermentable substrates profoundly limits microbial fermentation. Both extremes, uninterrupted infusion and prolonged nutrition deprivation, may disrupt the generation of microbial metabolites that contribute to intestinal barrier integrity, mucosal immunity, systemic immune regulation, and bidirectional communication along the gut–brain axis [18,55]. The shift is even more evident, as a brief 12 h nutrient interruption [56] triggers an immediate immunometabolic fasting signal: the body increases ketones and bilirubin, while lowering insulin and insulin-like growth factor-1 (IGF-1); the combined effect has been interpreted as an enhanced cellular repair program. Harnessing this balance between preserving SCFA production and safely controlling fasting harmful mediators could therefore reduce systemic and CNS inflammation [57].

Brief macronutrient interruption triggers ketogenesis within hours. The resulting rise in β-hydroxybutyrate (BHB) acts as a metabolic switch that simultaneously blunts early catabolism and activates cellular repair programs [58,59], and as a signaling metabolite it also inhibits NLRP3 inflammasome activation, improves redox balance and helps protect neurons and glia. This is in contrast to what would happen with continuous nutrition, which sustains insulin and IGF-1 signaling, suppressing ketone utilization and potentially delaying adaptive stress responses [60]. However, its implementation in standard ICU practice requires careful evaluation of risks (such as underfeeding, glycemic variability, and tolerance) against potential benefits of autophagy and immunometabolic reprogramming [56].

This feeding–fasting cycle has a “double-edged sword” effect on the gut–brain axis and neuroinflammation. Short-term, regulated fasting can increase microbial diversity, promote autophagy, and reduce systemic inflammation [61]. During fasting, the scarcity of nutrients favors bacteria that use substrates directly from the host, such as mucin and epithelial glycans, resulting in a thinning of the mucus layer, diminished barrier function, and reduced colonization resistance [62]. As soon as feeding is reestablished, this abrupt influx of new substrate can facilitate the proliferation of opportunistic proteobacteria and enterobacteria, with the release of LPS and other pro-inflammatory mediators. These mediators can translocate into the systemic circulation, triggering the immune system, even within the nervous system, as microglial activity is upregulated and cytokine release in the brain is increased [63]. Consequently, although moderate fasting may temporarily enhance neuroimmune equilibrium, excessive or prolonged fasting, particularly in metabolically vulnerable or critically ill patients, may disrupt the gut ecosystem and exacerbate neuroinflammatory processes due to this rebound “risk”.

The full set of mechanistic pathways is summarized in Table 1.

## 5. Ways to Interfere with the Gut–Brain Axis

From the pathways described above, the concept “microbiome-sparing feeding” could characterize feeding patterns and substrates that preserve saccharolytic commensals and their SCFA output. SCFAs come from the fermentation of dietary fibers and are the main fuel for colonocytes; they also influence microglial tone and the production of neuroactive metabolites and neurotransmitters such as serotonin and γ-aminobutyric acid (GABA)-related mediators that signal to the brain [30,67].

### 5.1. Changing the Diet: Fiber and Ketogenic Strategies

Diet composition, even before feeding timing, can shape the GM and its metabolic output. Fermentable fibers and prebiotic substrates give saccharolytic commensals more substrate and raise luminal SCFA production, which supports colonocyte metabolism, the mucosal barrier and a more regulatory immune tone [8,67]. However, fiber enrichment is not uniformly beneficial in the critically ill. A recent pilot randomized trial in critically ill trauma patients found that enteral supplementation with fermentable fiber (short-chain fructo-oligosaccharides) accelerated the loss of beneficial Bifidobacterium and Firmicutes and favored the expansion of pathogenic Enterobacteriaceae, with the microbial response shifting from beneficial to detrimental according to prior exposure to anaerobic antibiotics, underscoring that composition-based interventions are context-dependent [68]. Ketogenic diets work differently: they shift energy provision toward fat and sustain ketogenesis, reproducing some anti-inflammatory effects of fasting, including β-hydroxybutyrate-mediated inhibition of the NLRP3 inflammasome [60,69]. Both act through nutrient composition rather than timing, and are covered only briefly here because this review focuses on the temporal pattern of feeding (Section 5.2).

### 5.2. Non-Continuous, Time-Patterned Enteral Feeding

In the ICU, “fasting” is not a single intervention but a spectrum of intentional feeding modifications, from standard 24 h continuous EN to scheduled intermittent feeding pathways, to a complete halt of all nutrition support for a defined period. Each carries distinct metabolic and microbiological implications, and operational definitions are summarized in Table 2. The default gold standard has been, and remains, continuous feeding, where a 24 h infusion is maintained via a pump with no fasting interval [65]. The logic is simple, as it keeps the fuel coming.

Two of these descriptions are often used interchangeably, although they classify a schedule on different axes. Time-restricted or cyclic feeding is defined by when nutrition is given. Its window is anchored to the light–dark cycle and is intended to align peripheral clocks and microbial rhythmicity [70,71]; the overnight interval it creates may be too short to produce a metabolic fasting response at all. Fasting-mimicking is defined by what the interruption does metabolically: a cessation complete enough to lower insulin requirement and raise ketone bodies, at whatever hour it falls [56]. Interval length alone does not separate the two. A 10 h feeding window [71] entails a 14 h fast, longer than the 12 h interruption used in the fasting-mimicking pilot, and a 12 h/12 h nightly schedule satisfies both descriptions at once. The distinction matters for trial design. The two rationales call for different end-points, a metabolic readout for one and a rhythm readout for the other, and they carry different risks. Prolonged complete interruption entails hypoglycemia and a cumulative energy deficit [10,56] and, in brain-injured patients, the possibility of aggravating a cerebral metabolic crisis [72]. Compressing the same 24 h dose into a shorter window entails a higher hourly infusion rate and the gastrointestinal intolerance that follows from it [73,74].

Important boundaries and limitations that define the scope include exclusion criteria used in randomized controlled trials (RCTs) (e.g., those studies not including total parenteral nutrition, recent gastrointestinal (GI) surgery, or severe motility disorders), variation in fasting duration and position, some intermittent regimens having greater glycemic variability, and existing trials being underpowered for patient-centered outcomes [75].

Continuous EN is frequently interrupted in routine ICU care for procedures, airway management, medication administration, and gastrointestinal events, resulting in measurable caloric deficits (median three interruption episodes per patient; median interruption 5.5 h; interruptions accounted for ≈19% of designated EN time) [76]. Meal timing is a significant non-photic zeitgeber (a time cue that sets the body’s internal clock through eating patterns rather than light) for peripheral circadian clocks and directly influences metabolism and inflammation. Circadian regulation extends well beyond the central pacemaker. Peripheral molecular clocks located in the liver, intestinal epithelium, and immune cells are entrained predominantly by nutrient availability and feeding–fasting cycles through oscillations of the canonical CLOCK–BMAL1/PER–CRY transcriptional network. In parallel, the GM displays robust diurnal rhythmicity, with cyclical changes in microbial composition, gene expression, and metabolite production that are tightly coupled to host feeding behavior. This bidirectional synchronization between peripheral circadian clocks and the intestinal microbiome coordinates metabolic, epithelial, and immune functions, suggesting that disruption of feeding rhythms may perturb both microbial ecology and host circadian homeostasis [77]. Continuous 24 h enteral feeding in the ICU disrupts the body’s natural daytime-feeding/nighttime-fasting rhythm [78,79]. By contrast, intermittent or daytime-restricted feeding better mimics normal eating patterns and may preserve circadian alignment, hormonal rhythms, and metabolic functions that support recovery [77]. This argument concerns peripheral clocks. The suprachiasmatic nucleus is entrained principally by light: in healthy volunteers a five-hour delay in meal timing shifted peripheral metabolic rhythms without shifting the central melatonin and cortisol rhythms [79], and the same uncoupling of peripheral from master clock genes has been shown experimentally in sepsis [78]. Older observations bear directly on this. In eighteen patients in vegetative states fed enterally for four weeks, a daytime feeding window produced cortisol and core-temperature rhythms resembling those of healthy subjects, a night-time window shifted their peaks, and continuous feeding abolished them altogether [80,81]. Both are outputs that feeding itself can mask, melatonin was never measured, and the patients were not sedated under continuous light, so the finding falls short of demonstrating re-entrainment of the pacemaker. Under continuous ICU illumination and sustained sedation [65], no study has yet shown that a feeding schedule restores central rhythmicity. A randomized trial designed around exactly this question, taking the amplitude of the 24 h core body temperature rhythm as its primary outcome and melatonin among its secondary ones, completed recruitment in 2025 but has not yet reported [70]. Peripheral realignment should therefore not be read as recovery of the central clock, and the two should be assessed separately in any trial.

In addition to circadian effects, intermittent feeding activates fasting-type metabolic pathways—ketogenesis, reduced insulin/IGF-1, and autophagy induction—that can reshape the gut ecosystem and its metabolome [18,56]. Importantly, during short nutrient interruptions, the brain can rapidly use ketone bodies and lactate as alternative substrates to glucose, underscoring the importance of cerebral substrate safety when designing fasting schedules [82].

A feeding-time intervention presupposes that a circadian rhythm remains available to be entrained, and in the ICU this cannot be assumed. Critically ill adults are exposed to continuous artificial lighting, high noise levels and frequent care activities, and night-time artificial light may itself reduce melatonin production [83], while deep sedation further flattens the sleep–wake cycle. These act on the central pacemaker, whereas feeding time acts principally on peripheral clocks, so the environment may leave the peripheral signal without a central counterpart to align to and offset the intervention. Correcting the lighting alone has not proved sufficient [84]. Light exposure, sedation depth and sleep fragmentation should therefore be recorded as covariates, or used as stratification variables, in any trial of feeding timing in the ICU; a null result obtained without them cannot distinguish an ineffective intervention from an abolished rhythm.

**Table 2:** Operational definitions of feeding strategies used in studies investigating intermittent or time restricted nutrition in critical care and experimental models. Definitions are based on terminology reported in the cited literature.

Term	Operational Definition	Clinical Rationale	GM/Metabolic Effect	Key Evidence
Continuous feeding [85]	24 h pump infusion with no scheduled fasting interval	Standard of care; ensures consistent caloric and protein delivery; minimizes underfeeding risk in the acute phase.	Disrupts diurnal feeding/fasting cycle, attenuating circadian SCFA oscillation; sustains insulin/IGF-1 signaling, suppressing ketone utilization.	[65,85]
Intermittent feeding [59]	Multiple discrete feeds per 24 h (e.g., 4–6 or six feeds) creating fasting windows between boluses	Creates periodic fasting intervals between feeds; shortens time to energy target compared with continuous feeding.	Associated with flatter urea:creatinine ratio trajectory, suggesting reduced catabolism; no consistent GM diversity benefit across trials; increased diarrhea in largest RCT (11% vs. 4.7%); no muscle mass difference.	[10,55,75,86]
Cyclic/daily time-restricted feeding [71]	Nutrition delivered within a fixed daily window (e.g., 10 h feeding/14 h fast or 12 h/12 h) to create nightly fast	Delivers nutrition within a fixed daytime window to re-entrain peripheral circadian clocks and restore nocturnal fast.	Preserves diurnal microbial oscillation and circadian clock gene alignment (CLOCK, BMAL1); clinical GM data pending (DC-SCENIC results not yet published).	[65,66,71]
Bolus/postural intermittent feeding [87]	Large short-duration feeds delivered 3×/day often with right lateral tilt; creates long fasting intervals between feeds	Reduces aspiration risk via right lateral tilt; creates prolonged inter-meal fasting intervals.	Prolonged fasting intervals may activate mucin-scavenging taxa during nutrient scarcity; reduced aspiration indices vs. continuous in one RCT (8/56 vs. 26/51); no significant GI intolerance difference in multicenter RCT.	[87,88]
Fasting-mimicking (12 h interruption) [56]	Complete macronutrient cessation for 12 h, shown to induce ketogenesis and hormonal changes in ICU pilots (β hydroxybutyrate ↑, after 4 h—bilirubin ↑ and insulin/IGF-1 ↓ after 12 h)	Induces a metabolic fasting state to activate immunometabolic reprogramming; feasible in prolonged critical illness, with short-term tolerability but three episodes of severe hypoglycemia during the fasting interval and higher 90-day mortality in one crossover sequence.	12 h interruption confirmed: increased β-hydroxybutyrate, bilirubin; decreased insulin requirements and IGF-1; BHB inhibits NLRP3 inflammasome signaling; no detectable change in blood autophagy markers in pilot.	[56,60]
Sequential feeding [18]	Start continuous, transition to intermittent once caloric targets (e.g., 80%) met, then oral feeding	Balances early caloric adequacy (continuous phase) with later metabolic cycling (intermittent phase); progressive weaning toward oral intake.	Genus-level GM shifts (increased Erysipelotrichaceae_UCG-003, Howardella); improved albumin and lymphocyte counts; no change in Shannon alpha-diversity at Day 7; no difference in hospital mortality.	[18,89]
Early 72 h fasting [90] (observational definition)	No enteral/parenteral/oral nutrition for first 72 h after ICU admission (IV glucose possible)	Observational practice; not recommended for routine use by current guidelines; studied only in highly selected, severely ill patients.	Prolonged nutrient deprivation risks mucin-layer thinning and intestinal barrier dysfunction; refeeding after prolonged fast may trigger proteobacteria overgrowth; no mortality difference in propensity-matched cohort.	[85,90]

Abbreviations: BHB, β-hydroxybutyrate; BMAL1, brain and muscle ARNT-like 1; CLOCK, circadian locomotor output cycles kaput; GI, gastrointestinal; GM, gut microbiome; ICU, intensive care unit; IGF-1, insulin like growth factor 1; IV, intravenous; NLRP3, NOD-, LRR- and pyrin domain-containing protein 3; SCFAs, short-chain fatty acids.

### 5.3. Other Approaches to Microbiome Modulation

Beyond the timing of nutrient delivery, several other strategies can modulate the GM in critically ill and brain-injured patients. Probiotics and synbiotics aim to reintroduce beneficial commensals and have been associated with fewer episodes of ventilator-associated pneumonia and infection in a recent meta-analysis, although individual trials vary [91]. Prebiotic fibers and postbiotics, especially SCFAs, aim to restore microbial metabolites directly rather than the bacteria that make them [67]. Fecal microbiota transplantation works in refractory Clostridioide difficile infection and is being tested in ICU dysbiosis, but concerns about safety and standardization still limit its routine use [92]. Antibiotic stewardship and avoiding unnecessary microbiome-disrupting drugs are simpler, lower-risk measures [93]. All of these act on microbiome composition and complement feeding-pattern interventions rather than replace them; feeding pattern is the focus of this review. How these interventions interact with feeding pattern is largely unstudied. Nutrient timing determines when fermentable substrate reaches the colon, so an administered probiotic or a transplanted community encounters a different substrate environment depending on whether delivery is continuous or confined to a daytime window. Fasting intervals may favor mucin-foraging taxa at the expense of fiber fermenters, which is relevant both to probiotic persistence and to niche recovery after fecal microbiota transplantation. Whether feeding pattern modifies engraftment or the durability of these interventions has not been tested, and pairing a timing intervention with a microbiome-directed one is a reasonable next step.

## 6. Knowledge Gaps and Unanswered Questions

While these gut–brain interactions operate across all critically ill patients, their neurological consequences are most directly measurable and clinically significant in patients with primary or secondary brain injury. Nonetheless, evidence on how feeding timing affects the GM and host metabolic pathways derives predominantly from general ICU populations on mechanical ventilation. The few trials enriched for neurological phenotypes, discussed in Section 8.3, were designed around tolerance and delivery rather than around mechanism, and a multicenter trial of time-restricted enteral nutrition in severe stroke completed recruitment in 2025 without yet reporting [94]. Even the most recent synthesis of nutrition therapy in critically ill adults covers energy and protein dose, route of delivery and timing of initiation, but not the temporal pattern of delivery [95]. The following mechanistic and clinical evidence should therefore be read as the biological foundation upon which a dedicated, neurologically focused trial can be built.

Several questions remain unresolved. It is unclear whether the GM changes seen after brain injury cause neuroinflammation or merely follow it, and how much they reflect confounders such as antibiotic exposure [34], sedation and impaired gut motility rather than the injury itself [96]. The optimal feeding strategy for preserving the gut microbiome after acute brain injury remains undefined, with uncertainty extending to the timing, pattern, and duration of nutritional support. More importantly, current clinical research has largely failed to test the biological hypothesis underpinning microbiome-targeted nutrition. A multicenter randomized trial comparing time-restricted and continuous enteral feeding in severe stroke completed recruitment in 2025 and adopted 90-day functional recovery (modified Rankin Scale) as its primary endpoint. However, its results remain unavailable, and neither gut microbial ecology nor neuroinflammatory biomarkers were incorporated into the registered outcome measures [94]. Whether changing feeding pattern actually improves outcomes such as infection, delirium or neurological recovery is still unknown.

### Aim of This Review

This review brings together two strands of evidence that are usually kept apart: how the timing and pattern of EN affect the GM and host metabolism, studied almost entirely in general, mechanically ventilated ICU patients; and how the microbiome shapes neuroinflammation and recovery, studied mainly in observational and preclinical models of brain injury. No trial has yet tested feeding timing against microbiome or neuroinflammatory endpoints in patients with acute brain injury, and closing that gap is the central aim. The general-ICU feeding evidence is therefore used as the biological foundation, read through the lens of the gut–brain axis, to build a prioritized research agenda for the neurologically relevant ICU phenotypes in which a measurable benefit is most plausible.

## 7. Literature Search Strategy

### 7.1. Literature Search

A comprehensive search was performed in PubMed, Scopus, and Web of Science up to June 2026. The search combined free text terms and Medical Subject Headings (MeSH). Keywords included: intermittent feeding, intermittent fasting, time-restricted feeding, cyclic enteral nutrition, continuous enteral nutrition, GM, gut microbiome, dysbiosis, neuroinflammation, gut–brain axis, critical illness, intensive care unit, mechanical ventilation, ketogenic diet, ketogenesis, circadian rhythm, sepsis-associated encephalopathy, traumatic brain injury, delirium, and short-chain fatty acids. Boolean operators AND and OR were used to combine terms across domains. Reference lists of relevant articles were also screened to identify additional studies. The search strategies and the numbers of records retrieved are given in Tables S1 and S2.

### 7.2. Selection of the Studies Discussed

After duplicate removal, titles and abstracts were screened for relevance independently by two authors (A.C. and R.S.); disagreements were resolved by a third author (M.G.). Full texts were evaluated when necessary. Eligible articles included peer reviewed studies written in English that investigated intermittent feeding strategies or fasting related mechanisms involving the GM or neuroinflammatory pathways. Both preclinical and clinical studies were considered. Conference abstracts, editorials, and non-peer-reviewed material were excluded. Reports that could not be retrieved, and those excluded after full-text assessment, are listed with reasons in Table S3.

### 7.3. Data Extraction and Synthesis

Key information from selected studies included study design, population, experimental model, microbiome related findings, and reported neuroinflammatory mechanisms. Evidence was summarized narratively and organized according to mechanistic pathways linking feeding patterns, microbiome composition, and neuroinflammation. Preclinical evidence is reported before the clinical studies. Data were extracted without a standardized extraction form, and extracted values were not independently duplicated.

### 7.4. Quality Assessment

Because this work is a narrative review, no formal risk of bias assessment or meta-analysis was conducted. Studies were selected for their conceptual and mechanistic relevance to the topic. As a narrative review, the literature search was not prospectively registered in PROSPERO or equivalent databases. A structured appraisal of the methodological limitations of the included clinical studies is provided in Table S5.

## 8. Overview of the Retrieved Evidence

### 8.1. Scope of the Evidence Considered

**Figure 3:**
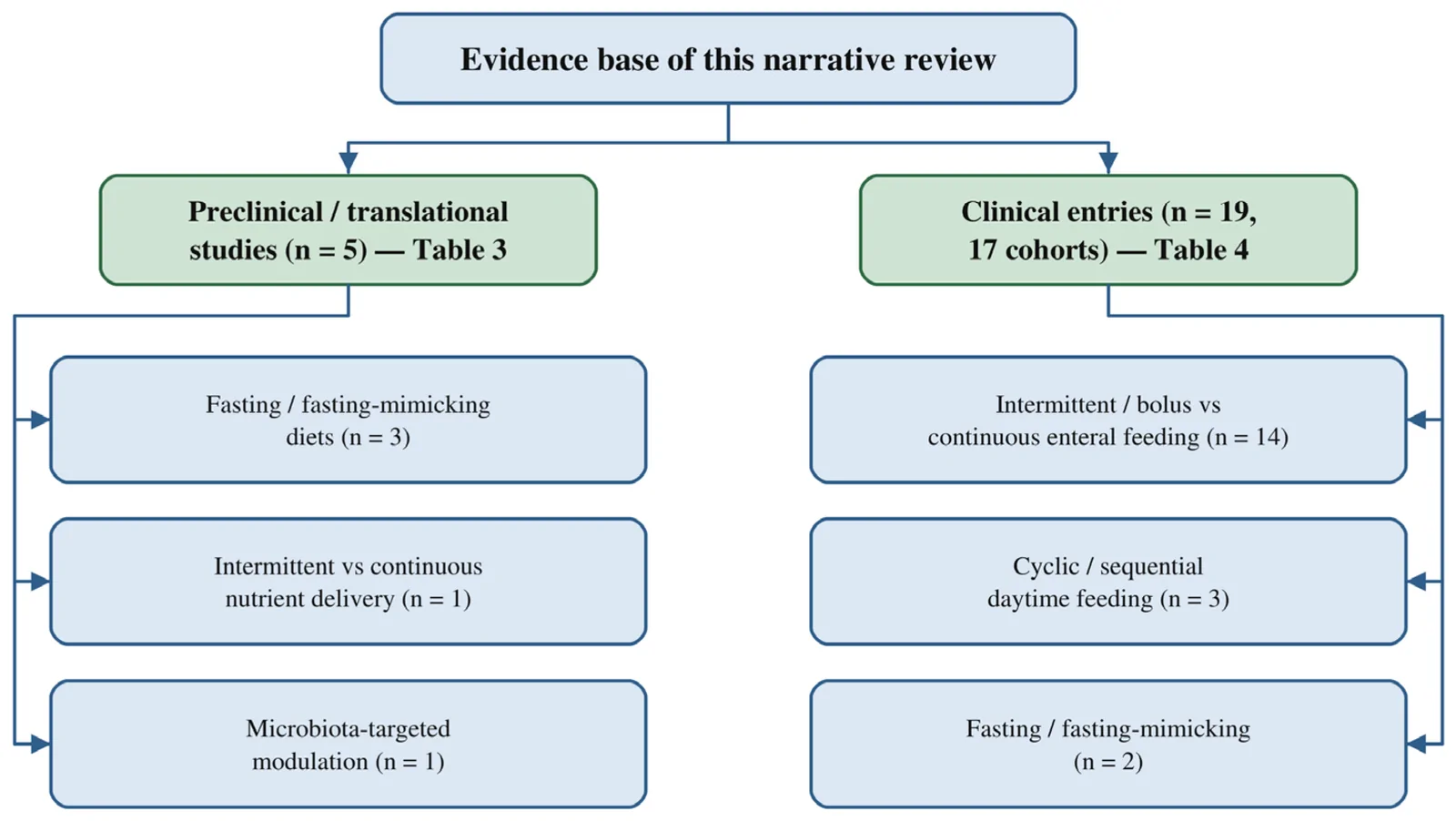
Overview of the evidence included in this narrative review, classified by study type (preclinical/translational versus clinical) and by intervention category. Counts refer to the studies discussed in Table 3 and Table 4. The search was not prospectively registered, and the figure is not a study-selection flow diagram. Table 4 lists nineteen entries: seventeen distinct clinical cohorts, one secondary analysis of a cohort already included and one registered trial that has not yet reported. Among the seventeen cohorts, one assessed gut microbiome endpoints, none measured neuroinflammatory biomarkers, one reported a neurological outcome measure (the Glasgow Coma Scale, as a secondary outcome), and five were dedicated brain-injured cohorts.

Figure 3 summarizes the evidence base of this review. It draws on five preclinical or translational studies (Table 3) and nineteen entries in Table 4, comprising seventeen distinct clinical cohorts, one secondary analysis of a cohort already included and one registered trial that has not yet reported, along with supporting pooled analyses. These covered continuous, intermittent, cyclic, bolus, sequential and fasting-mimicking enteral strategies as well as ketogenic and microbiome-targeted interventions. The preclinical evidence is presented first (Section 8.2), then the clinical evidence (Section 8.3). Figure S1 shows the flow of records; Tables S4 and S6 give the provenance of the included studies and the screening counts.

### 8.2. Preclinical Evidence

Preclinical experimental work in clinically relevant animal models supports several mechanistic pathways by which non-continuous feeding can alter host pathophysiology relevant to critical illness, including metabolic switching, autophagy induction, enhanced anabolic signaling, and microbiome-mediated organ protection. Key preclinical evidence is summarized in Table 3.

### 8.3. Clinical Evidence

Ketogenic diets (KDs) represent a composition-based, rather than timing-based, strategy to sustain endogenous ketogenesis by delivering the majority of calories as fat with minimal carbohydrate intake. In ICU patients with sepsis, a recent open-label RCT (*n* = 40) demonstrated that KD reliably induced stable ketosis, eliminated insulin requirements after Day 4 in all treated patients (vs. 35–60% in controls), and was associated with significantly more ventilation-free, vasopressor-free, and ICU-free days, without major metabolic complications [69]. Immunological analyses revealed reduced immune dysregulation with decreased T-cell activation gene expression, consistent with the known anti-inflammatory properties of β-hydroxybutyrate, including NLRP3 inflammasome inhibition [60]. No survival benefit was demonstrated at 30 days. While KD shares the key metabolic effectors of intermittent fasting—ketogenesis, reduced insulin/IGF-1, and NLRP3 inhibition—it operates through sustained metabolic reprogramming rather than cyclic fasting signals, raising distinct concerns about protein catabolism and electrolyte balance in prolonged critical illness. Clinical data in the ICU remain limited to small trials, and no study has yet integrated GM composition and neuroinflammatory endpoints as co-primary outcomes.

Most clinical trials available to date were conducted in general, mechanically ventilated ICU populations, and only five enrolled dedicated brain-injured cohorts. The findings below should therefore be read as a predominantly general-ICU foundation on which a neurologically focused study would build. On the clinical side, intermittent and cyclic feeding consistently induced fasting metabolic signals in the available trials—rising β-hydroxybutyrate, declining insulin requirement and IGF-1, and a flatter urea:creatinine ratio trajectory—with short-term tolerability reported in the ICU-FM-1 pilot crossover [56] and the sequential-feeding RCT [18]. That tolerability was not unqualified: severe hypoglycemia occurred in three patients during the fasting interval of the ICU-FM-1 crossover, in every case under intravenous insulin and in two after the insulin reduction prescribed by the protocol was omitted. A single-center randomized study in critically ill adults (*n* = 28) reproduced this cyclical pattern, with a higher peak plasma insulin concentration under diurnal intermittent gastric feeding than under continuous feeding (295.1 vs. 128.1 pmol/L) and no episode of hyperglycemia or hypoglycemia. However, diarrhea was again more frequent in the intermittent arm (5/11 vs. 0/17) [104]. However, these metabolic benefits have not translated into consistent patient-centered improvements. The most recent and largest RCT (*n* ≈ 294) found that intermittent feeding accelerated time to energy targets but increased diarrhea rates (11% vs. 4.7%) and had no effect on mortality, ventilator-associated pneumonia (VAP), or protein delivery [86], consistent with an earlier trial showing no benefit in muscle mass preservation despite higher protein delivery [55]. A pooled analysis of eight RCTs (*n* ≈ 993) confirmed no significant difference in hospital mortality (pooled risk ratio (RR) 0.97, 95% confidence interval (CI) 0.72–1.32) [75]. Two larger and more recent syntheses are less neutral. A meta-analysis of fifteen RCTs (*n* = 1406) reported more diarrhea (RR 1.52), more abdominal distension (RR 2.38), longer ICU stay and higher ICU mortality (RR 1.39, 95% CI 1.02–1.89) with intermittent feeding [74]. The same group’s update to twenty-two RCTs (*n* = 1662) confirmed the gastrointestinal findings but no longer detected a mortality difference [105]. In these pooled clinical-outcome data the signal therefore rests on gastrointestinal tolerance rather than on survival, and is most pronounced in mechanically ventilated patients. Notably, only one RCT to date incorporated GM end-points [18]; it found genus-level shifts and modest improvements in albumin and lymphocyte counts, but no change in alpha-diversity or hospital mortality. Untargeted serum metabolomics in the same trial identified 58 differentially abundant metabolites between the two arms, with primary bile acid biosynthesis the most enriched pathway. Specifically, 57.5% of that cohort had brain disease, and alpha-diversity did not differ within the brain-disease subgroup either. These genus-level shifts align with translational data linking dysbiosis and Proteobacteria overgrowth to enteral intolerance and systemic inflammatory signaling [66]. GM and neuroinflammation outcomes remain unmeasured in virtually all existing trials. Full details of individual studies are presented in Table 4.

A separate meta-analysis restricted to bolus versus continuous feeding, covering nine randomized trials published between 2020 and 2023 (*n* = 863), found no significant differences in gastrointestinal outcomes or length of stay. Both ICU and hospital mortality, however, approached significance in favor of continuous feeding (odds ratio [OR] 0.66, 95% CI 0.42–1.04 and OR 0.57, 95% CI 0.31–1.03), and seven of the nine trials carried a high risk of performance bias [73]. A three-arm randomized trial in intubated adults with sepsis (*n* = 93) extends that comparison to the feeding schedule itself. Against bolus and continuous feeding, an intermittent schedule with a nocturnal pause gave the most stable glucose profile and required insulin in the fewest patients (five of 31, against 11 of 31 and 15 of 31), although the incidence of high gastric residual volume did not differ between groups and mortality was not assessed [106].

Despite extensive investigation, no definitive conclusion could be drawn to favor one feeding mode over the other in the general ICU population [107]. This persistent lack of signal in heterogeneous cohorts points to the need to restrict future investigations to biologically informed subgroups, such as patients with neurological injury. The direct evidence in such cohorts is scarce, and it was built to answer a different question. Four studies compared bolus or intermittent with continuous delivery in head-injured, neurological-ICU, hemorrhagic-stroke and mixed neurosurgical populations, with samples of 34 to 152. Metabolic and delivery endpoints did not differ between schedules: respiratory quotient, resting energy expenditure and glycemia in ventilated head-injured adults [108], stool output, aspiration and caloric adequacy in a neurological ICU [109]. Where differences did emerge they favored continuous feeding, on tolerance and on delivery. Diarrhea affected 37.5% of intermittently fed patients with hemorrhagic stroke against 7.9% (*p* = 0.002) [110], and feeding intolerance 60.5% against 37.9% in the retrospective neurosurgical cohort [111], and continuous feeding also reached 75% of the nutritional goal faster in that cohort (median 3.3 vs. 4.6 days, *p* = 0.03). These are findings about tolerance and delivery. None of the four tested a fasting window and none measured microbiome or neuroinflammatory endpoints, so they constrain how a feeding schedule can be delivered safely after brain injury rather than testing whether the mechanism this review describes operates at all. The one study carrying a neurological outcome likewise tested something else. In tracheostomized adults with severe traumatic brain injury transferred from the ICU to a rehabilitation unit (*n* = 104), intermittent oro-esophageal feeding reduced aspiration pneumonia (OR 0.30, 95% CI 0.13–0.69) and produced a greater rise in the Glasgow Coma Scale (interaction β 0.98, 0.57–1.39) than nasogastric feeding, with route and schedule varied together in a post-acute setting and no fasting window [112].

**Table 4:** Summary of randomized and non-randomized clinical studies in critically ill adults comparing intermittent/cyclic/fasting-mimicking enteral feeding versus continuous 24 h feeding, reporting clinical, metabolic, and microbiome end-points. It is worth noting that, to date, only one study [18] has integrated microbiome end-points. Heterogeneous interventions, small sample sizes, and variable end-points limit pooled inference; pooled analyses reported increased diarrhea and modestly longer ICU stay with intermittent schedules in some RCTs, pilots demonstrate rapid metabolic fasting signals (↑ β-hydroxybutyrate, ↓ insulin), and secondary analyses suggest a flatter urea-to-creatinine catabolism trajectory with intermittent feeding, underscoring both potential benefit and risk and the need for adequately powered RCTs with integrated microbiome and patient-centered outcomes. No included study measured neuroinflammatory biomarkers. Figure 3 reports the neurological outcome measures and the brain-injury cohorts represented.

Study	Population	Intervention (Comparison)	Primary Endpoint(s)	Key Results	Negative or Null Findings and Limitations
**Hrdy et al., 2025—prospective randomized single-center trial **[86]	Critically ill adults at high nutritional risk; *N* = 300 randomized, 294 analyzed (INT 146 vs. CONT 148)	Intermittent EN (tolerance-driven) vs. Continuous EN (18 h/day protocol)	Time to reach ≥80% energy target	Intermittent shortened time (*p* = 0.009); no protein-target difference (*p* = 0.129). No statistically significant difference in 28-day mortality (continuous 31.1% vs. intermittent 27.4%)	No difference in 28-day mortality (continuous 31.1% vs. intermittent 27.4%) or in protein target achievement (*p* = 0.129); higher diarrhea incidence with intermittent feeding (11.0% vs. 4.7%, *p* = 0.049); single center.
**Sequential vs. Continuous feeding RCT (Qingdao Univ)—microbiome trial—2025 **[18]	Critically ill ICU patients expected to need prolonged enteral feeding (>10 days); mixed diagnoses (brain disease, sepsis predominance); ITT/analyzed *N* = 134	Sequential/intermittent feeding (three daily feeding windows after initial CF) vs. continuous feeding	Primary: gut microbiome α-diversity (Shannon index) at day 7; secondary: taxonomic composition, metabolites, clinical markers	No difference in Shannon α-diversity at day 7. Sequential feeding altered genus-level composition (↑ Erysipelotrichaceae_UCG-003, Howardella), improved albumin/cholesterol and lymphocyte increases; safety and glycemic events similar. No difference in hospital mortality (sequential 9.2% vs. continuous 13.0%, *p* = 0.484). The cohort was 57.5% brain disease and 32.8% sepsis, and in the brain-disease subgroup the Shannon index likewise did not differ	Primary endpoint negative: no difference in Shannon α-diversity at day 7, including within the brain-disease subgroup that made up 57.5% of the cohort. Genus-level shifts are of uncertain clinical significance; no neurological or neuroinflammatory endpoints; no follow-up beyond hospital discharge.
**DC-SCENIC—trial protocol -2024 NCT05627167 (completed Feb 2025) Final results not yet published **[71]	Ventilated ICU adults initiated on invasive MV ≤ 24 h and expected MV ≥ 72 h (planned *N* = 318)	Daily cyclic daytime enteral feeding (10 h window) vs. continuous 24 h feeding	Primary: ΔSOFA at day 7; secondary: delivery/tolerance/metabolic and respiratory outcomes, 28-day mortality	Protocolized trial designed to test whether daytime cyclic feeding reduces organ failure; results pending	Protocol only; results not yet published, so no findings can be attributed to this trial.
**Panwar et al.—multicenter RCT (three-times-day postural feeding) 2024 **[87]	Mechanically ventilated adult ICU patients (*N* = 120)	Intermittent postural feeding 3×/day (right lateral tilt) vs. standard continuous gastric feeding	GI intolerance incidence (vomiting/diarrhea/constipation); secondary: mortality, LOS, ventilator outcomes	No significant difference in GI intolerance; numerically lower but non-significant hospital mortality in intermittent group; study underpowered for mortality and other patient-centered end-points	No difference in GI intolerance; underpowered for mortality and other patient-centered endpoints.
**Cardozo Júnior et al.—retrospective cohort (first 72 h fasting)—2023 **[90]	Medical ICU adults with ICU LOS ≥5 days (propensity matched cohorts *n* = 93 vs. 93)	No nutrition support for first 72 h vs. any early nutrition (oral/EN/PN) in first 72 h	Hospital mortality; secondary: ICU mortality, LOS, duration MV, infections	After propensity matching, no difference in hospital or ICU mortality, 90-day survival, or other major secondary outcomes; suggests withholding nutrition for first 72 h may be safe in very severe patients but limited by observational design	No difference in hospital or ICU mortality, 90-day survival or other major secondary outcomes; retrospective, observational design.
**Puthucheary—secondary analysis (UCR catabolism) 2022 **[59]	Subset of UK ICU patients from multicenter trial (*n* ≈ 121) with high illness severity	Intermittent feeding vs. continuous feeding (same trial arms)	Urea:creatinine ratio (UCR) trajectory as marker of catabolism	Intermittent feeding associated with a significantly flatter UCR trajectory (coefficient −0.245, *p* = 0.002), suggesting mitigation of catabolism; baseline imbalance and exploratory design limit causal inference	Secondary exploratory analysis of an existing trial; baseline imbalance limits causal inference; surrogate endpoint only.
**Ren et al. Single-center RCT—2021 **[89]	Critically ill ICU patients; *N* = 62 (SF 32 vs. CF 30)	Sequential Feeding (early CF → intermittent circadian) vs. Continuous Feeding	Mean blood glucose over 7 days (non-inferiority)	SF median 8.8 mmol/L vs. CF 10.7 mmol/L (Z = −2.079; *p* = 0.019)	Single center, *N* = 62; glycemic surrogate endpoint; no microbiome or neurological outcomes.
**ICU-FM-1 pilot randomized crossover (fasting-mimicking)—2020 **[56]	Prolonged critically ill patients randomized around ICU day 6–8 (*n* = 70); requiring ongoing organ support	12 h feeding interval vs. 12 h nutrient interruption (crossover)	Metabolic fasting signals (bilirubin, insulin requirement, β-hydroxybutyrate), autophagy markers, short-term safety	12 h nutrient interruption induced a metabolic fasting response: ↑ serum bilirubin, ↓ insulin requirement, ↑ BHB, ↓ IGF-1. Blood autophagy markers unchanged. Feasible; limited by crossover design and pilot size. Mortality at 7 days comparable between the two groups. 90-day mortality was higher in the feeding–fasting group than in the fasting-feeding group (*p* = 0.003)	Blood autophagy markers unchanged. Severe hypoglycemia (arterial glucose < 40 mg/dL) occurred in three patients during the 12 h fasting interval, all receiving intravenous insulin and two after the protocol-specified insulin reduction was omitted; all were corrected with parenteral glucose. 90-day mortality was higher in the feeding–fasting sequence than in the fasting-feeding sequence (*p* = 0.003), a signal that warrants caution despite the pilot size and crossover design.
**Mcnelly et al.—RCT 2020** [55]	Mechanically ventilated ICU adults with multi-organ failure; expected prolonged ICU stay; *N* = 121	Intermittent bolus enteral feeding (6×/24 h) vs. continuous 24 h pump feeding	Primary trial: rectus femoris muscle CSA change over 10 days; safety and nutrition delivery	No difference in muscle mass loss at 10 days. Intermittent feeding achieved higher protein/energy delivery (≥80%) but increased glucose variability; overall feasible and safe without functional benefit in early critical illness. No statistically significant difference in mortality.	No difference in muscle mass loss at 10 days; increased glucose variability with intermittent feeding; no functional benefit.
**Kadamani et al., 2014—pseudo-randomized trial (Australian Crit Care) **[113]	Mechanically ventilated ICU patients; *N* = 30 (CEN 15 vs. BEN 15)	Continuous EN (CEN) vs. Bolus EN (BEN)	Aspiration and GI complications (3 days)	No aspiration in either group; Constipation ↑ CEN 66.7% vs. BEN 20% (*p* = 0.025)	*N* = 30, pseudo-randomized; no aspiration in either arm; 3-day observation only.
**Maurya et al., 2011—randomized trial, head injury **[108]	Adult men with head injury requiring controlled mechanical ventilation in ICU; *N* = 40	Continuous feeding over 18 h/day vs. six 3-hourly bolus feeds over 18 h, both 30 kcal/kg/day with a 6 h night rest	Respiratory quotient and resting energy expenditure measured every 30 min over 24 h; blood glucose	Respiratory quotient and resting energy expenditure comparable between regimens at baseline and at every measurement over 24 h (all *p* > 0.05); blood glucose not different; feeding adequacy (energy intake/measured resting energy expenditure, EI/MREE) 105.7% vs. 105.3%	Entirely null: no difference in respiratory quotient, resting energy expenditure or glycemia in a dedicated head-injury cohort; *N* = 40, single center, men only; 24 h observation; no microbiome, neuroinflammatory or neurological outcome.
**MacLeod et al., 2007—prospective RCT (trauma ICU) **[114]	Critically ill trauma patients; *N* = 164 (INT 79 vs. CONT 81)	Intermittent bolus every 4 h (30–60 min) vs. Continuous drip	Time to reach goal volume & days at 100% goal (10 days)	Faster goal achievement (Kaplan–Meier, log-rank *p* = 0.01); days at 100% of goal 4 vs. 3 (95% CI 3.5–4.4 vs. 2.7–3.6). Overall mortality 6.5% (17 deaths), with a non-significant trend towards higher mortality in the intermittent arm (*p* ≈ 0.18)	No significant mortality difference, but the non-significant trend favored continuous feeding (*p* ≈ 0.18); endpoints limited to delivery metrics.
**Chen et al., 2006—RCT **[88]	Ventilated critically ill patients; *N* = 107 (INT 56 vs. CONT 51)	Intermittent NG (4–6 boluses/day) vs. Continuous NG feeding	Aspiration indices & gastric emptiness (Day 7); extubation (Day 21)	Aspiration pneumonia patch on chest X-ray: INT 8/56 vs. CONT 26/51 (*p* < 0.001; adjusted OR 0.146, 95% CI 0.062–0.413); sputum glucose positive 13/56 vs. 25/51 (*p* = 0.005); extubation by day 21 60.7% vs. 31.4% (*p* = 0.002); higher total intake with INT (*p* < 0.001)	2006; no microbiome or neurological endpoints; aspiration ascertained by chest X-ray patch and sputum glucose strip, criteria the authors acknowledge lack specificity.
**Zhu et al., 2020—randomized controlled trial, hemorrhagic stroke **[110]	Patients with hemorrhagic stroke in a neurosurgery department; *N* = 78 (intermittent 40 vs. continuous 38)	Intermittent pump feeding four times daily vs. continuous 24 h pump feeding	Feeding intolerance; efficiency of calorie intake	Diarrhea was less frequent with continuous feeding (7.9% vs. 37.5%, *p* = 0.002) and total intolerance was lower (63.2% vs. 85.0%, *p* = 0.027). Calorie intake did not differ over the first three days (*p* = 0.099) or in total (*p* = 0.597)	Favors continuous feeding in a dedicated brain-injured cohort; no advantage in calorie delivery for either arm; single center, convenience sample; no microbiome, neuroinflammatory or neurological outcome.
**Kocan & Hickisch, 1986—randomized trial, neurological ICU **[109]	Adults in a neurological intensive care unit; *N* = 34, convenience sample randomly assigned	Continuous versus intermittent enteral administration	Stool number and consistency; aspiration, assessed by blue dye in pulmonary secretions; caloric intake as a percentage of goal	No significant difference in stool number or consistency, in evidence of aspiration, or in caloric intake as a percentage of goal. Level of consciousness on the Glasgow Coma Scale did not correlate with the incidence of aspiration	Entirely null in a dedicated neurological ICU cohort; *N* = 34, single center, 1986; aspiration ascertained by blue dye, a method since abandoned; no microbiome or neuroinflammatory endpoints.
**Rhoney et al., 2002—retrospective cohort, neurological/neurosurgical ICU **[111]	Consecutive adults with acute brain injury in a neurological/neurosurgical ICU at a level 1 trauma and tertiary referral center; *N* = 152 (bolus 86 vs. continuous 66)	Bolus vs. continuous gastric feeding, regimen chosen by clinician preference rather than randomized	Feeding intolerance (abdominal examination and gastric residuals > 75 mL over 4 h); time to nutritional goal	Feeding intolerance more frequent with bolus feeding (60.5% vs. 37.9%, *p* = 0.009); continuous feeding reached 75% of the nutritional goal faster (median 3.3 vs. 4.6 days, *p* = 0.03); trend towards fewer infections with continuous feeding (*p* = 0.05)	Favors continuous feeding in a dedicated brain-injured cohort; retrospective and non-randomized, with regimen assigned by clinician preference; intracerebral hemorrhage and ischemic stroke were independent predictors of intolerance; no microbiome or neuroinflammatory endpoints.
**DINE-normal, single-center randomized open-label trial—2025 **[104]	Critically ill adults expected to need gastric enteral feeding >48 h; mixed 48-bed ICU; *N* = 30 randomized (INT 13 vs. CONT 17), 28 analyzed (INT 11)	Diurnal intermittent gastric feeding at 08:00, 13:00 and 18:00 (each over 30–60 min) vs. continuous feeding; 48 h	Peak plasma insulin within 3 h of the first intermittent feed on study day 2	Peak insulin 295.1 ± 167.8 vs. 128.1 ± 57.2 pmol/L (*p* < 0.001); glucose not different; no hyper- or hypoglycemia; more frequent bowel movements and diarrhea with intermittent feeding (5/11 vs. 0/17; *p* = 0.005); no difference in vomiting, aspiration, delayed gastric emptying or ileus.	Diarrhea more frequent with intermittent feeding (5/11 vs. 0/17; *p* = 0.005); no between-group difference in plasma metabolites; *N* = 28 over a 48 h intervention.
**Lv 2026—Randomized controlled trial, severe TBI** [112]	Tracheostomized adults with severe TBI (GCS < 8) transferred from ICU to an inpatient rehabilitation unit after 14–28 ICU days; *N* = 104 (1:1), complete day-28 data in 98	Intermittent oro-esophageal tube feeding (3–5 feeds/day, ≤500 mL/feed) vs. nasogastric feeding (every 2–3 h, <200 mL/feed); 28 days	Nutritional status (hemoglobin, albumin, prealbumin, body mass index [BMI]); secondary: aspiration pneumonia, decannulation, GCS	Group-by-time interactions favored oro-esophageal feeding for albumin (β 3.675, 95% CI 1.854–5.496), hemoglobin (β 5.272, 2.707–7.837), prealbumin (β 11.835, 6.623–17.047) and BMI (β 1.719, 0.868–2.569). Aspiration pneumonia OR 0.304 (0.133–0.693; *p* = 0.005); decannulation hazard ratio (HR) 5.556 (3.197–9.657; *p* < 0.001); GCS interaction β 0.981 (0.572–1.390). Route/schedule comparison in a post-acute setting, not a fasting window; no microbiome or neuroinflammatory endpoints.	No microbiome or neuroinflammatory endpoints; conducted in a post-acute rehabilitation unit rather than in acute neurocritical care; feeding route and schedule are confounded; single center.
**Goksu 2025—single-center randomized three-arm trial, sepsis—2025 **[106]	Intubated adults with sepsis; APACHE II 8–25, BMI 18.5–30, non-diabetic; *N* = 93 (31 per arm)	Bolus vs. intermittent enteral feeding with nocturnal pause vs. continuous feeding; 7 days	Blood glucose level; secondary: feeding intolerance, high gastric residual volume, time to caloric target	Most stable glucose within and between days with intermittent feeding; mean high-GRV rate 1.17 ± 0.41 (intermittent) vs. 2.08 ± 0.67 (bolus) vs. 1.71 ± 0.49 (continuous), *p* = 0.014. Insulin required in 5/31 (intermittent), 11/31 (bolus), 15/31 (continuous). Incidence of high GRV not different (bolus 38.71%, intermittent 19.35%, continuous 22.58%; *p* = 0.183); time to caloric target not different (*p* = 0.414); mortality not assessed.	Incidence of high gastric residual volume not different (*p* = 0.183); time to caloric target not different (*p* = 0.414); mortality not assessed; non-diabetic patients only.

Abbreviations: APACHE II, Acute Physiology and Chronic Health Evaluation II; BEN, bolus enteral nutrition; BHB, β-hydroxybutyrate; CF, continuous feeding; CEN, continuous enteral nutrition; CONT, continuous group; CSA, cross-sectional area; ΔSOFA, change in Sequential Organ Failure Assessment score; EN, enteral nutrition; BMI, body mass index; CI, confidence interval; EI, energy intake; GCS, Glasgow Coma Scale; GI, gastrointestinal; GRV, gastric residual volume; HR, hazard ratio; ICU, intensive care unit; IGF-1, insulin-like growth factor 1; INT, intermittent feeding group; ITT, intention to treat; MREE, measured resting energy expenditure; LOS, length of stay; MV, mechanical ventilation; NG, nasogastric; NCT, National Clinical Trial identifier; PN, parenteral nutrition; RCT, randomized controlled trial; SF, sequential feeding; SOFA, Sequential Organ Failure Assessment; TBI, traumatic brain injury; UCR, urea-to-creatinine ratio; *n ≈*, approximate sample size.

## 9. Discussion

### 9.1. Summary of Findings

The clinical and preclinical evidence reviewed here suggests that the timing and pattern of EN can influence the GM, its metabolites and host metabolic signaling in critically ill patients. Clinical studies show that intermittent and cyclic feeding induce a fasting-type metabolic response and reach caloric targets at least as well as continuous feeding, with no consistent effect on mortality, although the largest pooled analyses report more diarrhea, more abdominal distension and longer ICU stay with intermittent schedules. The one trial that included microbiome endpoints found measurable shifts in gut taxa and in the serum metabolome. Preclinical models point in the same direction, connecting feeding–fasting cycles to SCFA production, barrier integrity, immune tone and neuroinflammation. Most of this evidence, however, comes from general ICU populations rather than from patients selected for brain injury. Five studies did enroll dedicated brain-injured cohorts, but none tested a fasting window against a microbiome-mediated mechanism. Four compared bolus or intermittent with continuous delivery, with results that were null or favored continuous feeding, and the fifth compared intermittent oro-esophageal with nasogastric feeding after transfer out of the ICU.

### 9.2. Promising Preclinical Evidence but Heterogeneous Clinical Results

Direct clinical evidence that a chrononutritional feeding intervention improves neurological outcomes does not yet exist; the case for targeting brain-injured patients is, for now, mechanistic and observational. ICU populations are heterogeneous, and existing RCTs are underpowered, in part because most do not preselect for neurological phenotypes most likely to respond to GM-targeted interventions [75]. Phenotype-guided enrichment increases signal-to-noise for mechanistic biomarkers and may substantially reduce required sample sizes. The ICU phenotypes with the strongest mechanistic rationale for gut–brain-feeding interactions are summarized in Table 5. The strength of direct clinical evidence linking enteral feeding timing → microbiome/metabolome → objective neuroinflammation or improved neurological outcomes is uneven across these phenotypes, so each phenotype should be treated as a prespecified subgroup or mechanistic substudy in future RCTs rather than assumed to respond identically. No trial has yet shown that a change in microbiome composition translates into a clinical benefit; where taxonomic shifts and patient-centered outcomes have been measured in the same trial, the relationship between them has not been formally analyzed. Part of this heterogeneity may be procedural rather than biological. Table 2 defines continuous feeding as a 24 h infusion without a scheduled fasting interval, yet the largest randomized comparison delivered its continuous arm over 18 h/day [86], and a trial in ventilated head-injured adults applied an 18 h schedule with a 6 h night rest to both arms [108]. Where the control arm already contains a nightly fast, the contrast under test is not feeding with a fasting window against feeding without one, but two patterns that differ in how that window is distributed. A null result obtained under those conditions constrains the effect of feeding pattern less than its wording implies, and the exposure actually delivered in each arm should be reported explicitly in future trials.

#### Why Microbiome Findings Do Not Converge

Several features of the existing microbiome literature explain why its findings do not converge. Antibiotic exposure, the single largest determinant of ICU dysbiosis [11], is rarely standardized between arms and is reported inconsistently, so an intervention effect and an antibiotic effect cannot be separated. Sample sizes are small: microbiome sub-studies are typically nested in trials powered for clinical end-points [18], and the resulting cohorts are underpowered for compositional comparisons given the variance that ICU cohorts show. The timing of sampling differs widely, and because the acute-phase microbiome shifts within hours to days [11], a sample drawn on day 3 and one drawn on day 7 describe different states. Methods differ further in sequencing target, taxonomic resolution and the diversity indices reported [124], so nominally similar studies are not directly comparable. Finally, the comparator itself is heterogeneous, as discussed above. Until these sources of variation are harmonized, the absence of a consistent signal should not be read as evidence of no effect.

### 9.3. Strengths

The main strength of this review is that it brings together mechanistic, preclinical and clinical evidence from the nutrition, microbiome and neurocritical care studies, which are rarely read side by side. Treating feeding pattern as a modifiable determinant of the gut–brain axis gives a target that is biologically plausible and clinically actionable. The operational definitions in Table 2, the evidence tables and the implementation framework are meant to make the idea usable at the bedside and to help design future trials.

### 9.4. Limitations

This review also has limitations. Being a narrative review, it did not use a prospectively registered search protocol and is therefore open to selection bias. The clinical evidence is heterogeneous in interventions, populations and endpoints, mostly comes from small and underpowered studies, and, most relevant here, remains dominated by general ICU rather than brain-injured cohorts. The single dedicated severe traumatic brain-injury study was conducted after transfer out of the ICU and compared feeding routes rather than a fasting window, so conclusions about microbiome-mediated or neuroinflammatory effects in brain-injured patients remain an extrapolation and are hypothesis-generating. Antibiotic exposure, a major driver of dysbiosis, is reported inconsistently and limits causal inference. Finally, only one trial so far has included microbiome endpoints, which limits any firm conclusion about microbiome-mediated effects of feeding pattern.

### 9.5. Perspectives

Two claims must be kept apart in what follows. The first is that the mechanism is coherent: feeding pattern demonstrably alters microbial rhythmicity, metabolite output and host metabolic signaling, and each of these is separately linked to neuroinflammation. The second is that the intervention is clinically operable in brain-injured patients, which requires evidence of safety, tolerability and benefit that does not yet exist. Mechanistic coherence is an argument for conducting a trial; it is not a reason to change clinical practice.

#### 9.5.1. Microbiome-Sparing Feeding Concepts

“Starving the bad bugs” may be an approach. Still another would be not to starve them at all and feed them from the moment the patient is admitted to the ICU, remembering that among those bugs are also key GM players that provide a formidable, beneficial output for the host.

For this reason, a microbiome-maintenance microfeed (a continuous, ultra-low caloric “prebiotic drip”) is worth considering. It would be an isotonic infusion of low osmolality, given at very low volume comparable to trophic EN rates or lower, containing fermentable soluble fiber such as oligofructose or inulin, to provide substrate for SCFA producers without materially increasing systemic caloric load. The EDEN RCT in 2012 [125] already demonstrated that trophic, hypocaloric enteral feeding can maintain gastrointestinal tolerance and safety in early critical illness, using standard polymeric formulas at minimal rates to protect the gut without delivering significant calories. In contrast, the concept we suggest evolves this approach from a purely metabolic strategy to a microbiome-maintenance strategy. Rather than providing inert calories at low rates, a microfeed “prebiotic drip” would supply a minimal, isotonic infusion designed not to feed the host, but to sustain commensal SCFA-producing microbes. The final goal is therefore to preserve commensal fermenters during periods when full EN is unsafe, for instance, during hemodynamic instability when there is a high risk of bowel necrosis and gut ischemia due to compromised splanchnic perfusion [16,126]. This could slow down the otherwise swift transition from GM to Gut “Pathobiota” [11].

A variant would be to give time-restricted prebiotic meals: short, small boluses of fermentable glycans (oligosaccharide/resistant starch blends) delivered only during daytime windows, to synchronize the microbial clock and keep SCFA output steady without substantially altering the caloric target. This would also avoid increasing splanchnic blood flow demand at a time when perfusion is required elsewhere.

Beyond prebiotics, postbiotics themselves could be used to replace host-interrupted SCFA signaling during the fasting phase: a small amount of butyrate/acetate could be delivered enterally or rectally in selected patients to provide energy to the enterocytes, preserve barrier integrity, and modulate immune signals [127].

Enteral strategies (even minimal) should not be used during active gut hypoperfusion or when escalating vasopressor requirements are present without preclinical/early-phase safety data. Evidence linking early full feeding with harm in unstable patients motivated this recommendation [85]. Furthermore, fermentable substrates can induce osmotic diarrhea if osmolality or delivery rate is poorly matched to absorptive capacity [128], so gradual dose escalation and pump control are essential. The prebiotic microfeed concept described above is therefore proposed only for the stabilizing or post-acute phase, once vasopressor requirements are decreasing and splanchnic perfusion is being restored —not as a strategy during active hemodynamic compromise.

#### 9.5.2. A Proposed Phased Clinical Trial

Collectively, the available experimental and clinical data support a transition from observational research toward mechanism-based interventional studies. We propose that the next generation of randomized clinical trials should evaluate whether aligning enteral nutrition with physiological feeding–fasting cycles, through daytime intermittent or cyclic (time-restricted) feeding, can preserve gut microbial homeostasis and attenuate neuroinflammation following acute brain injury. Such studies should move beyond conventional nutritional endpoints and incorporate serial characterization of the gut microbiome, microbial metabolomics, circadian biomarkers, systemic and neuroimmune responses, and clinically meaningful neurological outcomes. Several randomized trials have recently completed recruitment, including studies of cyclic enteral nutrition in mechanically ventilated ICU patients [71], cyclic daytime feeding with the amplitude of circadian rhythms as its primary outcome [70], and time-restricted enteral nutrition in severe stroke with a 90-day functional endpoint [94].

Their results should be incorporated before the design proposed here is finalized, and that design should be aimed at what they will leave open, namely whether the microbiome mediates any effect of feeding pattern on neuroinflammation. Mediation analysis linking feeding pattern to microbial and metabolomic change and then to neuroinflammatory markers cannot be performed on the aggregate published data reviewed here, and should be prespecified as an analytic objective of this trial.

The optimal window to assess whether intermittent or cyclic enteral feeding modifies pathogenic mechanisms is after the early, unstable “acute” phase, i.e., in the post-acute/stabilization and early rehabilitation periods, rather than immediately at ICU admission. The optimal mechanistic sampling window is Days 5–14 [18] (commonly Day 7–10) to collect microbiome (stool/rectal swab), metabolomics (serum/urine for ketones, SCFAs, bile acids), insulin/IGF-1, inflammatory cytokines and biomarkers analyses (NfL, GFAP, S100B) [129,130], and markers of autophagy. A baseline (early 4–24 h status) would also be pivotal. The samples should be frozen at −80 °C within strict timeframes and should then be analyzed centrally to minimize batch effects.

Assessing effects in the post-acute period [131] (days 7–14), at ICU discharge, and during early post-ICU rehabilitation (30–90 days) will capture mechanistic and clinically meaningful outcomes (muscle mass, delirium/neurologic recovery, infection, ventilator-free days, and functional recovery).

The study would enroll adults requiring mechanical ventilation and EN, excluding those with major gastrointestinal diseases or diabetes. Randomization would be stratified by antibiotic exposure and admission category to control key confounders.

Safety monitoring would focus on glycemic control and feeding intolerance [124]. Mechanistic end-points would be linked to clinical outcomes, such as ventilator-free days or ΔSOFA, using mixed-effects and mediation models. A pilot phase would assess feasibility and refine power estimates, paving the way for a larger, fully powered trial integrating multi-omics and clinical data.

The neuroinflammatory biomarkers proposed here require cautious interpretation. Serum S100B and glial fibrillary acidic protein lose their predictive value when extracranial injuries are present, so a treatment effect cannot be separated from the burden of systemic trauma [132]. Circulating concentrations also depend on renal clearance, which alters neurofilament light chain without this reflecting neuroaxonal injury [133]. No fluid marker therefore maps one-to-one onto neuroinflammation. Future trials should pair them with an independent readout of cerebral function or structure, such as quantitative electroencephalography, evoked potentials or serial imaging, so that convergent movement across modalities rather than a single analyte supports the inference.

#### 9.5.3. Practical Implementation and Safety

**Figure 4:**
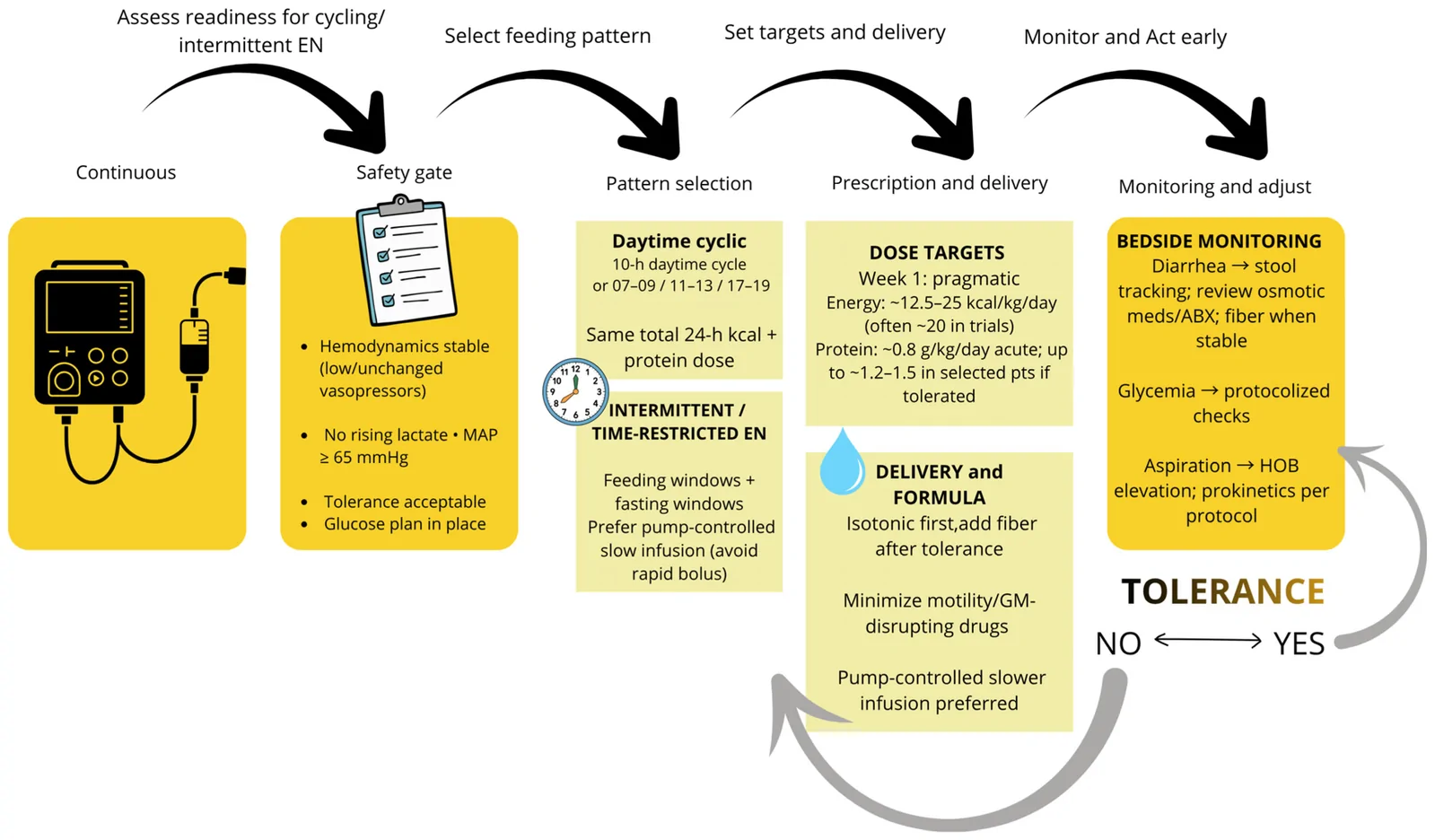
Practical framework for transitioning from continuous to cyclic or intermittent enteral nutrition in critically ill patients. The figure outlines a stepwise approach: (1) assessment of readiness for cycling with predefined safety criteria (hemodynamic stability, lactate trends, tolerance, and glucose control); (2) selection of feeding pattern (daytime cyclic or intermittent/time-restricted enteral nutrition) while maintaining the same 24 h energy and protein targets; (3) pragmatic prescription of dose targets, formula, and delivery method; and (4) early bedside monitoring with prompt adjustment based on tolerance. A feedback loop links tolerance assessment to feeding strategy refinement, emphasizing safety-driven, individualized implementation. Abbreviations: ABX, antibiotics; EN, enteral nutrition; GM, gut microbiome; HOB, head of bed; MAP, mean arterial pressure.

Implementing time-restricted or fasting-mimicking feeding strategies in the ICU requires careful patient selection and ongoing physiological assessment. These interventions should be initiated only after metabolic and hemodynamic stability has been achieved and should always be individualized according to the patient’s trajectory, ongoing therapies, and gastrointestinal tolerance; the readiness criteria are detailed in Table 6 and the clinical pathway is illustrated in Figure 4. These criteria align with current American Society for Parenteral and Enteral Nutrition (ASPEN) [134] and European Society of Intensive Care Medicine (ESICM) [135] recommendations for nutritional reintroduction following circulatory instability, and are intended to make the initiation of fasting intervals reproducible. They have not been validated as safe in brain-injured patients.

Non-continuous feeding should not be started during hemodynamic instability or an escalating vasopressor requirement, during an unresolved intracranial pressure crisis, or in patients receiving continuous intravenous insulin, in whom interrupting substrate delivery would create a mismatch between insulin and nutrient supply.

The criteria in Table 6 should be applied as a checklist and not combined into a score: they are expert-proposed thresholds, and weighting them would require either a cohort in which they are recorded against a defined early safety end-point or a formal consensus process.

Examples of daytime feeding protocols include 10 h daytime cycles or 07–09, 11–13, and 17–19 h feeding windows, while maintaining the same total 24 h caloric and protein dose. Caloric and protein guidance should follow contemporary ICU nutrition practice. Trials and protocols testing cyclic/diurnal strategies commonly used targets near 20 kcal/kg/day and ~0.8 g protein/kg/day in the acute phase [71]. Consensus guidance [85,138] supports a pragmatic kcal range during the first week of approximately 12.5–25 kcal/kg/day tailored to clinical context, and a higher protein prescription (up to ≈1.2–1.5 g/kg/day in some populations such as trauma) when tolerated and indicated.

Additionally, it would be advisable to deliver feeds via pump-controlled, slower infusions rather than rapid gravity/bolus pushes to reduce intolerance and aspiration risk where possible [139].

Intermittent feeding requires careful monitoring to prevent key complications. Diarrhea is a common issue [86] and should be managed with routine stool tracking, review of osmotic medications or antibiotics, and gradual introduction of soluble fiber once the patient is stable. Glycemic variability and hypoglycemia [56] must be prevented through protocolized glucose checks and insulin adjustments during fasting windows. Isotonic formulas should be preferred initially, with fiber [140] added only after tolerance is established, while minimizing drugs or antibiotics that impair motility or disrupt the GM. Aspiration risk remains variable across studies [88], so standard preventive measures, such as head elevation and the use of prokinetics, must be maintained at all times. A structured bedside monitoring plan is summarized in Table 7, outlining key surveillance domains and practical actions for early detection and management.

## 10. Conclusions

Intermittent or circadian-aligned feeding is a candidate strategy in critical care nutrition. If it restores physiological fasting–feeding rhythms, it offers a biologically coherent way to influence metabolism, GM, and brain–immune communication. The concept that short, time-gated nutrient restriction could “starve the bugs” (redirect a dysbiotic microbiome toward a healthier state) and “save the host” (limit neuroinflammation and organ dysfunction) is bold but mechanistically possible. Pilot studies have shown that short nutrient interruptions are feasible and are tolerated in selected patients. Feasibility, however, is not safety. Signals of harm have appeared both in individual trials and in recent pooled analyses. The studies conducted so far were small, only one was designed around a microbiome endpoint and none captured neuroimmune change, so clinical benefit remains unproven. The next generation of trials should couple metabolic and microbiome end-points with patient-centered outcomes to test whether time-patterned nutrition can truly reshape recovery in critical illness.

## Figures and Tables

**Table 1:** Key mechanistic pathways linking feeding–fasting cycles to gut–brain signaling. The pathways summarized here derive predominantly from preclinical models and from studies in chronic neurological disease; none have been demonstrated in acute brain injury, and the feeding-timing arm of each pathway acts on peripheral rather than central circadian control.

Pathway	Key Mediators	Fasting/TRF Effect	Excess Fasting Risk	Key Evidence
**Tryptophan-indole-kynurenine**	Indoles, kynurenines, AhR ligands	Preserved Treg/Th17 balance; neuroactive mediator production; barrier protection	Kynurenine shift may favor neurotoxic metabolites under prolonged deprivation	[64]
**Bile acids and lipid metabolites**	Secondary bile acids, TMAO, FXR/TGR5 ligands	Restored microbial bile acid biotransformation; reduced hepatic inflammation and TMAO	Bile acid pool depletion with prolonged fast may impair FXR signaling	[18,41]
**Barrier integrity and mucus**	Tight junction proteins, mucin, PAMPs	Restored SCFA-driven mucin synthesis; reduced LPS translocation and endotoxemia	Mucin degradation by host-substrate-scavenging taxa during nutrient scarcity	[41,57]
**Circadian synchronization**	CLOCK, BMAL1, PER/CRY, AMPK, mTOR	Re-entrainment of peripheral clocks; ketogenesis; anti-inflammatory gene programs	Circadian misalignment if feeding window is poorly timed relative to light cycle	[65,66]
**Vagal/autonomic anti-inflammatory reflex**	GM metabolites, vagal afferents, monoaminergic/GABAergic circuits	Preserved vagal tone; systemic and CNS anti-inflammatory reflex	Loss of vagal modulation with gut barrier disruption	[51]

Abbreviations: AhR, aryl hydrocarbon receptor; GABA, γ-aminobutyric acid; GM, gut microbiome; LPS, lipopolysaccharide; SCFAs, short-chain fatty acids; Treg, regulatory T cells; AMPK, AMP-activated protein kinase; BMAL1, brain and muscle ARNT-like 1; CLOCK, circadian locomotor output cycles kaput; CNS, central nervous system; FXR, farnesoid X receptor; mTOR, mechanistic target of rapamycin; PAMPs, pathogen-associated molecular patterns; PER/CRY, Period and Cryptochrome clock genes; TGR5, Takeda G-protein receptor 5; Th17, T-helper 17 cells; TMAO, trimethylamine N-oxide; TRF, time-restricted feeding.

**Table 3:** Key preclinical and translational evidence supporting the biological rationale for intermittent or fasting-mimicking nutrition strategies in critical illness.

Model/Context	Intervention	Key Mechanistic Pathway	Main Findings/Interpretation
Mouse (general preclinical FMD/fasting studies) [58]	Short fasts or fasting-mimicking diets (FMD)	Ketogenesis/metabolic switch; autophagy induction; anti-inflammatory signaling; organ protection	Fasting in mice, or FMD, produced a metabolic switch (ketogenesis), induced tissue autophagy with organ-specific timing, improved metabolic profiles, and demonstrated organ-protective effects in several models. These findings support the rationale for the ICU fasting trial.
Rat (rodent sepsis models) [97]	Microbiome modulation/taxa enrichment	Microbiome → host immunity/organ protection	Enrichment of certain taxa (e.g., *Erysipelotrichaceae UCG-003*) attenuated sepsis-induced lung injury in rat models, providing causal microbiome-mediated organ protection
Newborn pig (neonatal protein metabolism models) [98,99]	Intermittent/bolus protein vs. continuous infusion	Anabolic signaling (insulin + AA peaks); reduced protein catabolism; autophagy down-regulation	Bolus or intermittent protein delivery produced insulin/amino acid signaling peaks that enhanced muscle protein synthesis and lean mass compared to continuous infusion; intermittent feeding also reduced protein catabolism in pig models.
Murine tumor models (cancer) [100,101]	Fasting/FMD before or around chemotherapy	Differential stress resistance; metabolic reprogramming (glycolysis → ketones)	Several mouse studies have shown that fasting or FMD increases chemosensitivity and reduces tumor growth in some models, although the results were heterogeneous and sometimes neutral or harmful.
Mouse (organ-specific autophagy kinetics) [102,103]	Short fasting intervals	Tissue-specific autophagy induction timing	In mice, starvation-induced autophagy showed organ-dependent kinetics (e.g., early induction in muscle), indicating that blood markers may not accurately reflect tissue autophagy.

Abbreviations: FMD, fasting-mimicking diet; AA, amino acids; ICU, intensive care unit.

**Table 5:** Neurorelevant ICU phenotypes as targets for feeding-timing and microbiome interventions.

Phenotype	Key GM/Metabolic Link	Strength of Current Evidence	Priority End-Points
**Sepsis-associated encephalopathy**	Dysbiosis and microbial metabolites mechanistically implicated in BBB disruption, cytokine release, and brain dysfunction [115]	Moderate (observational + mechanistic)	Delirium-free days, ΔSOFA, SCFA/LPS levels
**Traumatic brain injury**	Rapid post-TBI gut dysbiosis with bidirectional neuroimmune signaling; antibiotic confounding prominent [116]	Moderate (preclinical strong; clinical exploratory)	Neuroinflammation biomarkers (NfL, GFAP), GM diversity
**Acute ischemic stroke/SAH**	GM shifts influence secondary brain injury and edema; nutrition timing potentially relevant [117,118]	Low-moderate (exploratory)	Functional outcome (mRS), inflammatory markers
**Post-cardiac arrest brain injury**	Global cerebral ischemia; ketogenesis and gut permeability may modulate secondary injury [119]	Low (mechanistic rationale only)	Ketone levels, neurological recovery scores
**Intracranial infection/encephalitis**	Central infections activate systemic and CNS immune responses likely modified by gut barrier integrity and GM-derived immune signals; microbiome/metabolome markers may be valuable [120]	Low (hypothesis-generating)	GM diversity, barrier markers, cytokine panel
**Acute delirium/encephalopathy of mixed etiology**	Common neuroinflammatory ICU phenotype sensitive to circadian feeding patterns, SCFA/ketone shifts, and gut–brain immune modulation [121]	Low-moderate (observational)	Delirium-free days, CAM-ICU, circadian biomarkers
**Prolonged disorders of consciousness/post-ICU cognitive impairment**	Ongoing gut–brain dysregulation during ICU may link to long-term cognitive deficits [122]	Low (hypothesis-generating)	90-day cognitive outcomes, GM/metabolome follow-up
**ICU-acquired muscle atrophy/critical illness myopathy**	Metabolic/inflammatory status shaped by feeding timing; feeding pattern affects protein anabolism [123]	Low-moderate (preclinical strong)	Muscle mass (ultrasound CSA), nitrogen balance

Abbreviations: BBB, blood–brain barrier; CAM-ICU, Confusion Assessment Method for the Intensive Care Unit; CNS, central nervous system; CSA, cross-sectional area; GFAP, glial fibrillary acidic protein; GM, gut microbiome; ICU, intensive care unit; LPS, lipopolysaccharide; mRS, modified Rankin Scale; NfL, neurofilament light chain; SAH, subarachnoid hemorrhage; SCFAs, short-chain fatty acids; SOFA, Sequential Organ Failure Assessment; TBI, traumatic brain injury.

**Table 6:** Checklist to implement intermittent nutrition. Note: The checklist synthesizes practical thresholds and should be interpreted within the overall clinical context and reassessed before each progression of fasting duration. Unless a source is cited, thresholds are expert-proposed operational criteria derived from general ICU evidence; none has been validated specifically in brain-injured patients, and the neurological criteria are proposed here for the purpose of a future trial rather than for current practice.

Domain	Criterion	Operational Threshold	Rationale
**Hemodynamic**	Vasopressor support	Stable or decreasing for ≥12–24 h; norepinephrine ≤ 0.05 µg/kg/min without recent escalation	Indicates restored macrocirculatory flow and oxygen delivery
	Serum lactate	≤2 mmol/L or consistently trending downward	Reflects resolution of tissue hypoxia and adequate perfusion
	Mean arterial pressure (MAP)	≥65 mmHg (with stable APP ≥ 60 mmHg if available)	Surrogate of splanchnic perfusion and intestinal viability
	Urine output	≥0.5 mL/kg/h for ≥6 h	Reflects renal perfusion and overall circulatory stability
	Absence of signs of hypoperfusion	Warm extremities, normal capillary refill, decreasing vasopressor index	Clinical indicators of restored tissue flow
**Gastrointestinal**	Enteric sounds	Present in ≥2 quadrants	Suggests preserved motility and vagal activation
	Gastric residual volume (GRV)	≤500 mL/6 h where GRV is measured, as a tolerance check before opening a fasting window rather than as routine monitoring; no vomiting or distension	Acceptable tolerance threshold per ESICM/ASPEN guidelines [134,135]; routine GRV monitoring is not recommended in current practice, and the 500 mL threshold follows REGANE [136]
	Bolus or intermittent trial	50–100 mL bolus tolerated without regurgitation or discomfort	Confirms readiness for cyclic/bolus administration
	Abdominal perfusion pressure (APP)	≥60 mmHg (if monitored)	Ensures gut mucosal perfusion before fasting intervals
	Absence of bowel ischemia or ileus	No new distension, pain, or high residuals	Prevents enteral intolerance during fasting windows
	Gastroparesis	No escalation of prokinetics and no repeated high residuals in the preceding 24 h	Impaired gastric emptying precludes intermittent delivery
**Metabolic**	Glycemic control	100–160 mg/dL (5.5–8.8 mmol/L) without severe hypoglycemia in prior 12 h	Ensures metabolic flexibility before fasting initiation
	Acid–base status	pH ≥ 7.35, base deficit improving or ≤4 mmol/L	Excludes ongoing anaerobic metabolism
	β-hydroxybutyrate	<2 mmol/L unless intentional in fasting-mimicking regimen	Avoids uncontrolled ketosis or substrate deficit
	Electrolytes (K^+^, Mg^2+^, P)	Within normal range and stable for ≥12 h	Prevents arrhythmias or refeeding-like instability
	Inflammatory and nutritional trend	CRP decreasing; nitrogen balance ≥ −5 g/day if available	Reflects systemic recovery and tolerance potential
**Neurological**	Cerebral perfusion pressure (CPP)	≥60 mmHg where intracranial pressure is monitored	Lower bound of the 60–70 mmHg target recommended for severe traumatic brain injury [137]; maintains cerebral oxygen delivery across the fasting window
	Intracranial stability	No intracranial pressure crisis or neurological deterioration in the preceding 24 h	Avoids opening a fasting window during unstable intracranial physiology
	Glycemic variability	Coefficient of variation < 30% over the preceding 24 h	Glucose excursions have been implicated in secondary brain injury

Abbreviations: APP, abdominal perfusion pressure; ASPEN, American Society for Parenteral and Enteral Nutrition; CPP, cerebral perfusion pressure; ESICM, European Society of Intensive Care Medicine; CRP, C-reactive protein; GRV, gastric residual volume; MAP, mean arterial pressure.

**Table 7:** Clinical monitoring checklist for intermittent or fasting-mimicking feeding in the ICU. This table summarizes the key domains for bedside monitoring once intermittent feeding is initiated. The monitoring targets listed are expert-proposed operational criteria and have not been validated specifically in brain-injured patients.

Domain	Key Checks	Practical Notes
**Diarrhea**	• Review recent antibiotics, osmotically active drugs (e.g., lactulose, sorbitol), magnesium-containing preparations, and enteral formulations. • Exclude *Clostridioides difficile* if ≥3 loose stools/day. • Adjust fiber and prebiotic content according to stool frequency.	Replace hyperosmolar formulas; consider *soluble fiber* or peptide-based formulas; evaluate need for *slow-infusion restart* after fasting windows.
**Glycemia**	• Set target 100–160 mg/dL (5.5–8.8 mmol/L). • Perform capillary glucose checks before and after the fasting window. • Reduce the insulin infusion pre-emptively when the fasting interval begins, as prescribed in the ICU-FM-1 protocol [56]; the severe hypoglycemic events in that trial occurred when this reduction was omitted.	Re-evaluate insulin requirements after each cycle; monitor for rebound hyperglycemia upon refeeding.
**General tolerance**	• Document feeding-related symptoms (nausea, discomfort, distension). • Record stool frequency and consistency daily. • Track residual volumes and abdominal pressure.	Any deterioration should prompt return to continuous feeding and reassessment of stability criteria.

Abbreviations: ICU, intensive care unit; ICU-FM-1, first intensive care unit fasting-mimicking pilot trial.

## Data Availability

No new data were created or analyzed in this study. Data Sharing is not applicable to this article.

## Supplementary Materials

The following supporting information can be downloaded at https://www.mdpi.com/article/10.3390/nu18172907/s1, Table S1. Search strategies, as executed; Table S2. Records retrieved and duplicates removed; Table S3. Reports not retrieved, and reports excluded after full-text assessment; Table S4. Search-yield check: provenance of the clinical studies included in Table 4; Table S5. Structured appraisal of the methodological limitations of the included clinical studies; Table S6. Screening counts for the final stage of the search; Figure S1. Flow of records through the documented search.

## Abbreviations

AA	amino acids
AhR	aryl hydrocarbon receptor
AMPK	AMP-activated protein kinase
APP	abdominal perfusion pressure
ASPEN	American Society for Parenteral and Enteral Nutrition
BBB	blood–brain barrier
BEN	bolus enteral nutrition
BHB	β-hydroxybutyrate
BMAL1	brain and muscle ARNT-like 1
CAM-ICU	Confusion Assessment Method for the ICU
CEN	continuous enteral nutrition
CF	continuous feeding
CI	confidence interval
CLOCK	circadian locomotor output cycles kaput
CNS	central nervous system
CONT	continuous group
CRP	C-reactive protein
CRY	Cryptochrome clock genes
CSA	cross-sectional area
EI	energy intake
EN	enteral nutrition
END	enteral nutrition-related diarrhea
ESICM	European Society of Intensive Care Medicine
FFAR	free fatty acid receptor
FMD	fasting-mimicking diet
FXR	farnesoid X receptor
GABA	γ-aminobutyric acid
GFAP	glial fibrillary acidic protein
GI	gastrointestinal
GM	gut microbiome
GRV	gastric residual volume
HPA	hypothalamic–pituitary–adrenal axis
HR	hazard ratio
ICU	intensive care unit
IGF-1	insulin-like growth factor-1
IL	interleukin
INT	intermittent feeding group
ITT	intention to treat
IV	intravenous
LOS	length of stay
LPS	lipopolysaccharide
MAP	mean arterial pressure
MODS	multi-organ dysfunction syndrome
MREE	measured resting energy expenditure
mRS	modified Rankin Scale
mTOR	mechanistic target of rapamycin
MV	mechanical ventilation
NF-κB	nuclear factor kappa B
NfL	neurofilament light chain
NG	nasogastric
NLRP3	NOD-, LRR- and pyrin domain-containing protein 3
NO	nitric oxide
OR	odds ratio
PAMPs	pathogen-associated molecular patterns
PER	Period clock genes
PN	parenteral nutrition
RCT	randomized controlled trial
ROS	reactive oxygen species
RR	risk ratio
SAE	sepsis-associated encephalopathy
SAH	subarachnoid hemorrhage
SCFAs	short-chain fatty acids
SF	sequential feeding
SOFA	Sequential Organ Failure Assessment
TBI	traumatic brain injury
TGF-β	transforming growth factor-beta
TGR5	Takeda G-protein receptor 5
Th17	T helper 17 cells
TLR4	Toll-like receptor 4
TMAO	trimethylamine N-oxide
Treg	regulatory T cells
TRF	time-restricted feeding
UCR	urea-to-creatinine ratio
VAP	ventilator-associated pneumonia
