# Supplementary material for: Improving the Outcome of Brain-Injured Patients by Non-Continuous Feeding to Prevent Dysbiosis
Source: Nutrients. 2026 Sep 4;18(17):2907. doi: 10.3390/nu18172907 (PMC13566997; doi:10.3390/nu18172907)
Supplement: Supplementary file 1 [file nutrients-18-02907-s001.zip › nutrients-4544519-supplementary.pdf]

Supplementary Materials

This document reports the literature search underlying the present narrative review in a form that allows it to be reproduced independently. It is provided in response to reviewer requests for database-specific search strategies, execution dates, applied limits, record counts and reasons for exclusion.

The scope of what is claimed. The present work is a narrative review. Reporting the search in this detail documents what was searched; it does not assert exhaustive retrieval, and no compliance with PRISMA or any other systematic-review reporting standard is claimed. The search was not prospectively registered, as already stated in Section 7.4 of the main text.

Table S1. Search strategies, as executed

Database	Q1	Q2	Q3	Sum of the three queries	Records after within-database de-duplication	Duplicates removed within database
PubMed	359	97	356	812	807	5
Scopus	487	170	654	1311	1300	11
Web of Science	357	176	544	1077	1067	10
Total	1203	443	1554	3200	3174	26

Coverage cut-off: June 2026 (unchanged from the original submission). Execution date of the queries reported below: 23 August 2026. The strategies were re-executed on that date so that record-level yields could be documented; the coverage cut-off was not changed.

Three concept blocks were used, corresponding to the three strands the review brings together: (Q1) feeding pattern in critical illness; (Q2) gut microbiome and feeding pattern in critical illness; (Q3) fasting or ketogenic interventions and neuroinflammation.

Databases: PubMed, Scopus, Web of Science Core Collection. Two blocks of the PubMed strategy used MeSH descriptors (“critical illness”, “enteral nutrition”). Scopus and Web of Science do not index MeSH, so in those databases the same concepts were expressed as title/abstract/keyword phrases. Date limiting also differs between platforms: PubMed allows a day-level limit (to 30 June 2026), whereas Scopus and Web of Science were limited at the year level (publication year ≤ 2026).

PubMed

```
Q1  ("intermittent feeding"[tiab] OR "intermittent fasting"[tiab] OR "time-restricted feeding"[tiab] OR "time restricted feeding"[tiab] OR "cyclic feeding"[tiab] OR "cyclic enteral nutrition"[tiab] OR "bolus feeding"[tiab] OR "continuous feeding"[tiab] OR "continuous enteral nutrition"[tiab]) AND ("critical illness"[MeSH Terms] OR "critically ill"[tiab] OR "intensive care"[tiab] OR "ICU"[tiab] OR "mechanical ventilation"[tiab] OR "enteral nutrition"[MeSH Terms])
```

```
Q2  ("gut microbiome"[tiab] OR "gut microbiota"[tiab] OR dysbiosis[tiab] OR "short-chain fatty acids"[tiab] OR "gut-brain axis"[tiab]) AND ("enteral nutrition"[tiab] OR "enteral feeding"[tiab] OR "intermittent fasting"[tiab] OR "time-restricted feeding"[tiab] OR "feeding pattern"[tiab] OR "bolus feeding"[tiab]) AND ("critically ill"[tiab] OR "intensive care"[tiab] OR ICU[tiab] OR sepsis[tiab] OR "brain injury"[tiab])
```

```
Q3  ("intermittent fasting"[tiab] OR "time-restricted feeding"[tiab] OR "ketogenic diet"[tiab] OR ketogenesis[tiab] OR "caloric restriction"[tiab]) AND (neuroinflammation[tiab] OR "traumatic brain injury"[tiab] OR "sepsis-associated encephalopathy"[tiab] OR delirium[tiab] OR "acute brain injury"[tiab])
```

Scopus

Q1 TITLE-ABS-KEY("intermittent feeding" OR "intermittent fasting" OR "time-restricted feeding" OR "time restricted feeding" OR "cyclic feeding" OR "cyclic enteral nutrition" OR "bolus feeding" OR "continuous feeding" OR "continuous enteral nutrition") AND TITLE-ABS-KEY("critical illness" OR "critically ill" OR "intensive care" OR "ICU" OR "mechanical ventilation" OR "enteral nutrition") AND PUBYEAR < 2027

Q2 TITLE-ABS-KEY("gut microbiome" OR "gut microbiota" OR "dysbiosis" OR "short-chain fatty acids" OR "gut-brain axis") AND TITLE-ABS-KEY("enteral nutrition" OR "enteral feeding" OR "intermittent fasting" OR "time-restricted feeding" OR "feeding pattern" OR "bolus feeding") AND TITLE-ABS-KEY("critically ill" OR "intensive care" OR "ICU" OR "sepsis" OR "brain injury") AND PUBYEAR < 2027

Q3 TITLE-ABS-KEY("intermittent fasting" OR "time-restricted feeding" OR "ketogenic diet" OR "ketogenesis" OR "caloric restriction") AND TITLE-ABS-KEY("neuroinflammation" OR "traumatic brain injury" OR "sepsis-associated encephalopathy" OR "delirium" OR "acute brain injury") AND PUBYEAR < 2027

Web of Science Core Collection

Q1 TS=("intermittent feeding" OR "intermittent fasting" OR "time-restricted feeding" OR "time restricted feeding" OR "cyclic feeding" OR "cyclic enteral nutrition" OR "bolus feeding" OR "continuous feeding" OR "continuous enteral nutrition") AND TS=("critical illness" OR "critically ill" OR "intensive care" OR "ICU" OR "mechanical ventilation" OR "enteral nutrition")

Q2 TS=("gut microbiome" OR "gut microbiota" OR "dysbiosis" OR "short-chain fatty acids" OR "gut-brain axis") AND TS=("enteral nutrition" OR "enteral feeding" OR "intermittent fasting" OR "time-restricted feeding" OR "feeding pattern" OR "bolus feeding") AND TS=("critically ill" OR "intensive care" OR "ICU" OR "sepsis" OR "brain injury")

Q3 TS=("intermittent fasting" OR "time-restricted feeding" OR "ketogenic diet" OR "ketogenesis" OR "caloric restriction") AND TS=("neuroinflammation" OR "traumatic brain injury" OR "sepsis-associated encephalopathy" OR "delirium" OR "acute brain injury")

In addition to the database searches, reference lists of retrieved articles and of relevant reviews were hand-searched, as stated in Section 7.1 of the main text. Records identified in that way are accounted for separately in Table S4.

Table S2. Records retrieved and duplicates removed.

Stage	n
Records returned by the search strategies (sum of the three databases)	3,200
Duplicates removed within each database, by the database's own record identifier	26
Records remaining	3,174
PubMed records that no longer resolve, excluded as not characterizable	4
Records entering the cross-database comparison	3,170
Duplicates removed on DOI (n = 1,407) or on title with agreement on year (n = 30)	1,437
Unique records	1,733

Arithmetic check: 3,170 − 1,437 = 1,733.

Note. Within-database duplicates arise because the three concept blocks overlap—an article indexed under both intermittent fasting and gut microbiome is returned by more than one query. De-duplication was performed on the exported records by exact match on the normalized DOI (lower-cased, prefix stripped) and, where a DOI was absent, by normalized-title match with agreement on publication year to within one year. Records without a DOI: PubMed 43, Scopus 84, Web of Science 59. Pairwise overlap between databases: PubMed and Scopus 696, PubMed and Web of Science 618, Scopus and Web of Science 712; 596 records were returned by all three. Four PubMed records returned by the searches no longer resolve to a retrievable record and could not be characterized; they are reported as identified but excluded from the comparison rather than silently dropped. The de-duplication script and the merged record list are available from the corresponding author.

Table S3. Reports not retrieved, and reports excluded after full-text assessment.

The counts below cover the stages for which the selection process was recorded at the time. For earlier stages of the search, decisions on individual full-text reports were not logged separately, and no figures are reported for them here.

Report	Category	Reason
Gungor 2011	Not retrieved	Published in a journal not indexed in the databases searched; no retrievable record
Wang 2011	Not retrieved	Published in a journal not indexed in the databases searched; no retrievable record
Chen 2017	Not retrieved	Published in a journal not indexed in the databases searched; no retrievable record
Mazaherpour 2016	Not retrieved	Citation could not be verified against any indexed record
Taylor 1982	Excluded after assessment	No abstract and insufficient reported data to characterize the intervention
Suzuki 2022	Excluded after assessment	Compares rate of administration, not feeding pattern
ESO guideline 2026	Excluded after assessment	Clinical practice guideline, not a primary study
Two reviews	Excluded after assessment	Review articles, not primary studies
Two preclinical studies	Excluded after assessment	Preclinical; already covered by Table 3 of the main text
One clinical study	Excluded after assessment	Addressed nutrient composition rather than the timing of delivery

Note. Four reports were sought but could not be retrieved; eight were assessed and not retained. Reasons are stated at the level at which the decision was actually made.

Table S4. Search-yield check: provenance of the clinical studies included in Table 4

This table reports, for each clinical study included in Table 4 of the main text, whether it is retrieved by the documented search strategies or was identified through other methods (reference-list hand-searching and targeted verification). It is provided so that the adequacy of the search can be assessed directly rather than inferred from record counts.

Study (reference in main text)	In the PubMed record set	In the merged three-database set
Yao 2025	Yes	Yes
McNelly 2020	Yes	Yes
Van Dyck, ICU-FM-1 2020	Yes	Yes
Flower / Puthucheary 2022	Yes	Yes
Hrdy 2025	Yes	Yes
Panwar 2024	Yes	Yes
Chen 2006	Yes	Yes
Ren 2021	Yes	Yes
Cardozo Júnior 2023	No	No
DINE-normal / Beattie 2025	Yes	Yes
Goksu 2025	Yes	Yes
Maurya 2011	Yes	Yes
Kocan & Hickisch 1986	No	No
Zhu 2020	No	Yes
Rhoney 2002	No	No
Lv 2026	No	No
Kadamani 2014	Yes	Yes
MacLeod 2007	Yes	Yes

Note. Fourteen of the eighteen clinical studies included in Table 4 are returned by the documented search strategies; thirteen are returned by the PubMed strategies alone, and Zhu 2020 is added by Scopus and Web of Science. The four not returned were identified through reference-list hand-searching and targeted verification: Cardozo Júnior 2023, Kocan & Hickisch 1986, Rhoney 2002 and Lv 2026. Kocan & Hickisch 1986 predates consistent electronic indexing of the terminology used in the strategies. Matching was performed on the normalized DOI of each included study against the merged record set.

Table S5. Structured appraisal of the methodological limitations of the included clinical studies.

Consistent with the narrative design of this review, no formal risk-of-bias instrument was applied and no summary score is reported. The appraisal below is organized by the methodological features that determine how far the clinical evidence in Table 4 can be taken, and states, for each, which studies are affected and what follows for interpretation.

Domain	What the included studies show	Consequence for interpretation
Population	No trial enrolled a dedicated acute brain-injury cohort as its primary population apart from five studies (Kocan & Hickisch 1986; Rhoney 2002; Maurya 2011; Zhu 2020; Lv 2026); the remainder are general, mostly mechanically ventilated ICU populations.	Conclusions about neurological benefit are extrapolations and remain hypothesis-generating.
Blinding	Feeding-pattern interventions cannot be masked from bedside staff; no included trial achieved blinding of care providers, and outcome assessment was rarely blinded.	Tolerance and gastrointestinal outcomes, which depend on bedside judgement, are the outcomes most exposed to performance and detection bias.
Sample size	Most trials enrolled fewer than 150 participants; several fewer than 50.	Individual trials are underpowered for mortality and for infrequent harms; pooled estimates dominate, and are themselves sensitive to which trials are included.
Definition of the comparator	"Continuous" feeding is not defined identically across trials: at least one trial delivered the continuous arm over 18 h/day, and another allowed a 6 h nocturnal rest in both arms.	Some contrasts labelled continuous versus intermittent compare two intermittent schedules, which biases towards the null and may explain part of the negative findings.
Endpoints	Of the seventeen distinct clinical cohorts, one assessed gut microbiome endpoints, none measured neuroinflammatory biomarkers, and one reported a neurological outcome measure (Glasgow Coma Scale, as a secondary outcome).	The mechanistic chain proposed in this review has not been tested end-to-end in any clinical study; the causal step from microbiome change to neurological outcome remains untested.
Design	The evidence base mixes randomized trials, one crossover pilot, retrospective cohorts and one secondary analysis of an already-included cohort.	Observational entries are subject to confounding by indication: patients fed continuously may differ systematically in severity from those fed intermittently.
Reporting	Some trials report percentages without denominators, or report a p-value in the abstract that differs from the one in the results section.	Where source figures were internally inconsistent, counts rather than percentages are reported in Table 4, and the discrepancy is stated.

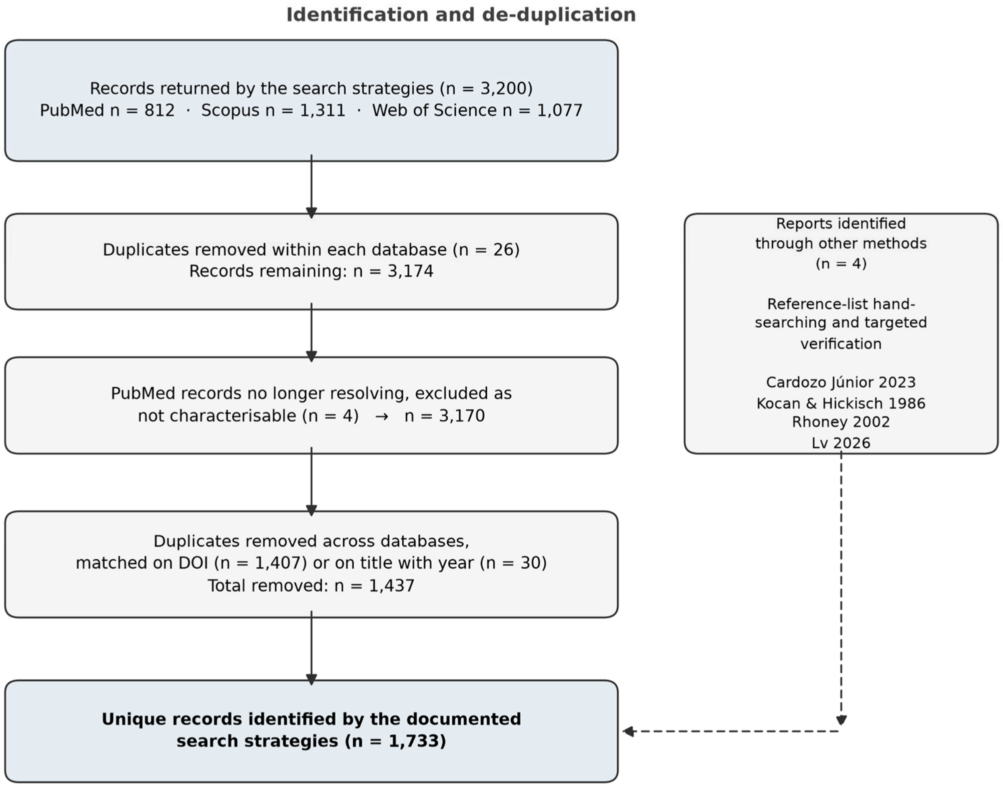

Figure S1. Flow of records through the documented search. Records returned by the three search strategies were de-duplicated first within each database using the database's own record identifier, then across databases by exact match on the normalized DOI or, where a DOI was absent, by normalized-title match with agreement on publication year. Four PubMed records returned by the searches no longer resolve to a retrievable record and could not be characterized; they are shown as identified but excluded from the comparison. The broken arrow denotes reports identified through other methods, namely reference-list hand-searching and targeted verification. Of the eighteen clinical studies included in Table 4 of the main text, fourteen are returned by the documented strategies; the four shown here were identified through other methods, and their provenance is reported in Table S4. The diagram documents identification and de-duplication, which were recorded. Stage-by-stage screening counts were not logged at the time the review was conducted and are therefore not shown; presenting counts reconstructed after the fact would give figures that were never recorded the standing of a contemporaneous record. Counts are available for the final stage of the search, which brought coverage to the June 2026 cut-off, and are reported in Table S6. This figure is not a study-selection flow diagram for a systematic review, and Figure 3 of the main text is a different object: it summarizes the evidence base that was included.

Table S6. Screening counts for the final stage of the search.

Record-level screening counts exist for the final stage of the search, which brought coverage to the June 2026 cut-off. They are reported here in full. For the earlier stages, screening was not logged record by record, and no counts are reported for them.

Stage	n
Records returned by the targeted query (randomized trials and meta-analyses of feeding patterns in critically ill adults)	8
Records returned by the broader query (gut microbiome, dysbiosis, short-chain fatty acids and the gut–brain axis in critical illness)	91
Records screened on title and abstract	99
Reports assessed in full text	9
Included in Table 4	3
Cited elsewhere in the text (Section 6)	1
Excluded after full-text assessment	5

Note. The five reports excluded after full-text assessment were two review articles, two preclinical studies, and one study addressing nutrient composition rather than the timing of delivery; they are listed in Table S3. The three studies included at this stage were the DINE-normal proof-of-concept study, the randomized trial of intermittent oro-oesophageal feeding in severe traumatic brain injury, and the three-arm trial of glycemic control in sepsis.
